# Automatic multispectral MRI segmentation of human hippocampal subfields: an evaluation of multicentric test–retest reproducibility

**DOI:** 10.1007/s00429-020-02172-w

**Published:** 2020-11-24

**Authors:** Andrea Chiappiniello, Roberto Tarducci, Cristina Muscio, Maria Grazia Bruzzone, Marco Bozzali, Pietro Tiraboschi, Anna Nigri, Claudia Ambrosi, Elena Chipi, Stefania Ferraro, Cristina Festari, Roberto Gasparotti, Ruben Gianeri, Giovanni Giulietti, Lorella Mascaro, Chiara Montanucci, Valentina Nicolosi, Cristina Rosazza, Laura Serra, Giovanni B. Frisoni, Daniela Perani, Fabrizio Tagliavini, Jorge Jovicich

**Affiliations:** 1 Department of Physics, University of Turin, Turin, Italy; 2 Medical Physics Department, Ospedale Santa Maria della Misericordia, Piazzale Giorgio Menghini 1, 06129 Perugia, Italy; 3 Division of Neurology V/Neuropathology, Fondazione IRCCS Istituto Neurologico Carlo Besta, Milan, Italy; 4 Unit of Neuroradiology, Fondazione IRCCS Istituto Neurologico Carlo Besta, Milan, Italy; 5 Neuroimaging Laboratory, Fondazione IRCCS Santa Lucia, Rome, Italy; 6 Department of Neuroscience, Brighton and Sussex Medical School, University of Sussex, Brighton, UK; 7 Neuroradiology Unit, ASST Spedali Civili Di Brescia, Brescia, Italy; 8 Laboratory of Clinical Neurochemistry, Neurology Clinic, Center for Memory Disturbances, University of Perugia, Perugia, Italy; 9 Laboratory of Neuroimaging and Alzheimer’s Epidemiology, IRCCS Istituto Centro San Giovanni Di Dio Fatebenefratelli, Brescia, Italy; 10 Department of Molecular and Translational Medicine, University of Brescia, Brescia, Italy; 11 Department of Medical and Surgical Specialities, Radiological Sciences and Public Health, University of Brescia, Brescia, Italy; 12 Nuclear Medicine Unit, IRCCS San Raffaele Hospital, Milan, Italy; 13 Department of Diagnostic Imaging, Medical Physics Unit, ASST Spedali Civili Di Brescia, Brescia, Italy; 14 Laboratory of Neuroimaging of Aging, LANVIE, University of Geneva, Geneva, Switzerland; 15 Memory Clinic, University Hospital, Geneva, Switzerland; 16 Division of Neuroscience, IRCCS San Raffaele Scientific Institute, Milan, Italy; 17 Vita-Salute San Raffaele University, Milan, Italy; 18 Scientific Direction, Fondazione IRCCS Istituto Neurologico Carlo Besta, Milan, Italy; 19 Center for Mind/Brain Sciences (CIMeC), University of Trento, Rovereto, Italy

**Keywords:** Hippocampal subfields, Automated segmentation, FreeSurfer, Test–retest reproducibility, Human brain morphometry, Multispectral MRI

## Abstract

Accurate and reproducible automated segmentation of human hippocampal subfields is of interest to study their roles in cognitive functions and disease processes. Multispectral structural MRI methods have been proposed to improve automated hippocampal subfield segmentation accuracy, but the reproducibility in a multicentric setting is, to date, not well characterized. Here, we assessed test–retest reproducibility of FreeSurfer 6.0 hippocampal subfield segmentations using multispectral MRI analysis pipelines (22 healthy subjects scanned twice, a week apart, at four 3T MRI sites). The harmonized MRI protocol included two 3D-T1, a 3D-FLAIR, and a high-resolution 2D-T2. After within-session T1 averaging, subfield volumes were segmented using three pipelines with different multispectral data: two longitudinal (“long_T1s” and “long_T1s_FLAIR”) and one cross-sectional (“long_T1s_FLAIR_crossT2”). Volume reproducibility was quantified in magnitude (reproducibility error—RE) and space (DICE coefficient). RE was lower in all hippocampal subfields, except for hippocampal fissure, using the longitudinal pipelines compared to long_T1s_FLAIR_crossT2 (average RE reduction of 0.4–3.6%). Similarly, the longitudinal pipelines showed a higher spatial reproducibility (1.1–7.8% of DICE improvement) in all hippocampal structures compared to long_T1s_FLAIR_crossT2. Moreover, long_T1s_FLAIR provided a small but significant RE improvement in comparison to long_T1s (*p* = 0.015), whereas no significant DICE differences were found. In addition, structures with volumes larger than 200 mm^3^ had better RE (1–2%) and DICE (0.7–0.95) than smaller structures. In summary, our study suggests that the most reproducible hippocampal subfield FreeSurfer segmentations are derived from a longitudinal pipeline using 3D-T1s and 3D-FLAIR. Adapting a longitudinal pipeline to include high-resolution 2D-T2 may lead to further improvements.

**Electronic supplementary material:**

The online version of this article (10.1007/s00429-020-02172-w) contains supplementary material, which is available to authorized users.

## Introduction

The human hippocampal formation is a complex brain structure widely studied by neuroscientists due to its involvement in different cognitive processes, such as episodic memory (Squire et al. 1992; Gorbach et al. 2017) and spatial navigation (Maguire et al. 1998; Eichenbaum 2017), in verbal memory (Ezzati et al. 2016; Zammit et al. 2017), in normal development (Gogtay et al. 2006; Sussman et al. 2016), in adult age-related changes (Mueller et al. 2007; Fjell et al. 2014), as well as in neurological and psychiatric pathology, including Alzheimer’s disease (AD) (Dubois et al. 2007; Mufson et al. 2015), epilepsy (Bernasconi et al. 2011; Winston et al. 2017), autism (Aylward et al. 1999; Barnea-Goraly et al. 2014), bipolar disorders (Moorhead et al. 2007; Mamah et al. 2016), and schizophrenia (Levitt et al. 2010; Kalmady et al. 2017). In particular, there is a very high interest in characterizing and relating structural and functional reorganization of the hippocampus (Zarei et al. 2013; Przeździk et al. 2019).

Beyond the interest in the anatomical segmentation of the whole hippocampal formation, in the last decade, magnetic resonance imaging (MRI) morphometry has highlighted that hippocampal subfields may allow for more accuracy than the whole hippocampus in detecting pathological changes (Pluta et al. 2012; La Joie et al. 2013). Manual segmentation is still considered the gold standard, but it has two main challenges: cost (i.e. time to acquire the expertise and time to perform the segmentations) and variability across raters (Nugent et al. 2007; Lee et al. 2015; Yushkevich et al. 2015b). These limitations have stimulated interest in developing automated tools for the segmentation of hippocampal subfields. Several studies have compared different automated tools with manual segmentations to evaluate segmentation accuracy, and FreeSurfer is considered among the most accurate methods (Tae et al. 2008; Morey et al. 2009; Zanfidar et al. 2017).

FreeSurfer (http://surfer.nmr.mgh.harvard.edu/, RRID: SCR_001847) allows the automatic delineation of hippocampal subfields from structural MRI data using different segmentation options. FreeSurfer requires a 3D-T1 for brain segmentation, but optional images may be also used, such as additional 3D-T1, high-resolution 2D-T2, and 3D-FLAIR data. The updated algorithm released in FreeSurfer 6.0 for the segmentation of hippocampal subfields uses a new atlas constructed from ultra-high resolution ex-vivo MRI (Iglesias et al. 2015), producing sub-hippocampal volume estimates that better match histological data. The latest version of FreeSurfer has shown higher hippocampal segmentation accuracy (Iglesias et al. 2015) relative to segmentations obtained in earlier FreeSurfer versions (Pluta et al. 2012; de Flores et al. 2015). One of the factors related to the segmentation improvement is the use of a high-resolution 2D-T2 acquisition perpendicular to the hippocampus, which adds a different contrast that is particularly relevant in the delineation of the molecular layer. In particular, Mueller et al. (2018) have shown that hippocampal subfield segmentation approaches that involve high-resolution T2 images outperformed those using only the whole-brain T1 images in the detection of early stage atrophy and in association with amyloid positivity and general cognitive performance. Moreover, a longitudinal module specific for the segmentation of the hippocampal subfields was implemented in FreeSurfer 6.0 (Iglesias et al. 2016). Currently, this module does not allow the use of additional high-resolution 2D-T2 images.

In addition to the high-resolution 2D-T2 images that help hippocampal segmentation, it is also possible to conduct a multispectral segmentation of gray matter using fluid attenuation inversion recovery (FLAIR) images. The use of 3D-FLAIR data can improve the separation of gray matter tissue from pial, vessels and extracerebral connective tissue at brain edges (Viviani et al. 2017). It has been seen that the combined use of 3D-T1 and 3D-FLAIR data specifically improves the segmentation accuracy in the medial and inferior faces of the hippocampal regions, areas in which there is an interest in detecting atrophy (Viviani et al. 2017). Therefore, altogether these findings motivate the interest in considering 3D-T1, 3D-FLAIR and high-resolution 2D-T2 for improved automated hippocampal subfields.

Besides the challenge of obtaining accurate automated segmentations of the hippocampal subfields, an additional issue is related to the reproducibility of these segmentations, particularly in clinical multicentric longitudinal MRI studies. Segmentation reproducibility may be evaluated by doing repeated experiments in a short time using healthy subjects (i.e. test–retest acquisitions, Morey et al. 2010). The current literature presents very few test–retest 3T studies evaluating FreeSurfer hippocampal subfield segmentations in healthy subjects, as outlined in Table 1. In particular, Marizzoni et al. (2015) used 3T scanners and FreeSurfer version 5.1, demonstrating that using the average of two within-session 3D-T1 acquisitions significantly improves test–retest reproducibility of hippocampal subfields. Other studies used single 3D-T1 acquisition and a variety of software versions (Whelan et al. 2016; Worker et al. 2018; Brown et al. 2020). However, none of these studies evaluated how the use of multispectral data from the same session, such as 3D-FLAIR or high-resolution 2D-T2, may affect the reproducibility of hippocampal subfield segmentations with respect to the use of a single T1 contrast.

**Table 1:** Summary of the studies that evaluated test–retest reproducibility of hippocampal subfield volume segmentations derived from FreeSurfer using structural MRI data

Study	MRI scanners (number of sites)	Healthy subjects (years age range)	Within-session structural MRI data acquisition			FreeSurfer version	FreeSurfer stream for hippocampal subfield reproducibility analysis
			3D-T1	3D-FLAIR	2D-T2		
This study	Multisite 3T study: Philips Achieva (3), Siemens Skyra (1)	22 (25–65)	Two	One	One	Longitudinal whole brain: 6.0 Hippocampal subfields: 6.0	Longitudinal, averaged T1 Longitudinal, averaged T1 + FLAIR Cross-sectional, averaged T1 + FLAIR + T2
Brown et al. (2020)	Upgrade study: 3T Siemens Trio (1) to 3T Siemens Prisma (1)	11 (22–55)	Two	–	–	Longitudinal whole brain: 6.0 Hippocampal subfields: 6.0	Longitudinal, single T1 (the best of the two acquired)
Worker et al. (2018)	3T GE Discovery (1)	22 (50–73)	One	–	–	Longitudinal whole brain: 5.3 Hippocampal subfields: 6.0	Longitudinal, single T1
Whelan et al. (2016)	3T GE (1)	163 (68–80)	One	–	–	Cross-sectional whole brain: 5.3 Hippocampal subfields: 6.0	Cross-sectional, single T1
Marizzoni et al. (2015)	Multisite 3T study: Siemens Allegra (1), Siemens TIM Trio (2), Siemens Verio (1), Siemens Skyra (1), Siemens Biograph (1), GE HDxt (2), GE Discovery (1), Philips Achieva (4)	65 (50–80)	Two	–	–	Longitudinal whole brain: 5.1 Hippocampal subfields: 5.1	Cross-sectional, single and averaged T1

Since the focus of this 3T study is the test–retest reproducibility on healthy subjects, the table reports the data associated with 3T acquisitions of healthy volunteers, even if some studies also evaluated other populations (Worker et al. 2018; Whelan et al. 2016)

Currently there is little consensus on whether the use of multispectral MRI data may affect the reproducibility of hippocampal subfield volumes, particularly in longitudinal multicentric studies using automatic segmentation tools. Accordingly, the aim of this study is to evaluate the reproducibility of the hippocampal subfield volume segmentations, using three FreeSurfer segmentation pipelines based on multispectral structural 3T MRI data: two 3D-T1, one 3D-FLAIR, and one high-resolution 2D-T2 anatomical volume. To the best of our knowledge, there are no publicly available datasets containing this complete set of multispectral brain anatomical data for the evaluation of test–retest data in healthy subjects. In particular, the ADNI3 (http://adni.loni.usc.edu) and Human Connectome Project (HCP, https://www.humanconnectome.org) MRI protocols do not consider two 3D-T1 acquisitions, the HCP and PharmaCog (https://www.alzheimer-europe.org/Research/PharmaCog) MRI protocols do not provide a 3D-FLAIR, while high-resolution 2D-T2 was not acquired in the PharmaCog MRI protocol. Thus, the images were acquired in four different 3T MRI centers using a harmonized MRI protocol implemented within the Italian AD-NET project, a multicentric initiative focused on the development of operational research criteria for early recognition of typical and atypical forms of AD integrating clinical, imaging, and molecular data. Exploiting FreeSurfer 6.0, we evaluated the hippocampal subfield segmentation reproducibility in three different pipelines: two longitudinal pipelines (with and without FLAIR images) and the cross-sectional pipeline with FLAIR and high-resolution T2 images. The optimal pipeline will be applied on the longitudinal MRI data of the clinical population acquired within this multicentric initiative to characterize the atrophy progression in the hippocampus and its subfields.

## Materials and methods

### Participants

A total of 22 subjects with no history of neurological, psychiatric, or cognitive impairment disorders participated in this study that involved four clinical 3T MRI sites across Italy (Milan, Perugia, Brescia, and Rome). Subjects underwent two MRI sessions approximately a week apart. Table 2 summarizes basic demographics, MRI site, and scan interval information. Written informed consent as approved by the local Ethics Committees from each participating Institution was provided by each volunteer.

**Table 2:** Summary of subjects’ demographic data and main MRI system information across sites

	Subjects’ age: mean ± SD (years)	Subjects’ gender (males/females)	Test–retest interval: mean ± SD (days)	3T MRI scanner	MR system software version	Receiver coil channels
Site1: IRCCS “Carlo Besta”, Milan	55 ± 11	6/1	6.9 ± 0.4	Philips Achieva	5.1	32
Site 2: Perugia University, Perugia	49 ± 8	3/2	7 ± 0	Philips Achieva	2.6.3	8
Site 3: Brescia University, Brescia	50 ± 8	3/2	7.2 ± 0.4	Siemens Skyra	Numaris4 syngo MR E11	64
Site 4: IRCCS “Santa Lucia”, Rome	36 ± 9	3/2	11 ± 7	Philips Achieva	3.2	32

### MRI scanners and acquisition protocol

The main specifications of the different 3T clinical MRI scanners are reported in Table 2. The MRI protocol was harmonized using only vendor-provided sequences and keeping the following main parameters for the various structural sequences. The acquisition protocol for each test and retest sessions included a 3D sagittal T1-weighted sequence (FOV 240 × 240 mm^2^, 180 slices, voxel size 1 × 1 × 1 mm^3^, TE 3.9 ms, FA 8°, no fat suppression, and no averages, approximate acquisition time 4 min 30 s) at the beginning and another one at the end of the scanning session, a 3D axial FLAIR T2-weighted sequence (FOV 240 × 240 mm^2^, 180 slices, voxel size 1 × 1 × 1 mm^3^, TI 1650 ms, fat suppression, 2 averages, approximate acquisition time 5 min), and a 2D high-resolution coronal T2-weighted sequence covering the whole hippocampus with slices oriented perpendicular to its main anterior–posterior axis (FOV 200 × 200 mm^2^, 60 slices, voxel size 0.4 × 0.4 × 2 mm^3^, TE 120 ms, no fat suppression, 2 averages, approximate acquisition time 6 min). The overall acquisition protocol lasted about 50 min because it included other sequences. The choice of acquisition parameters of the various sequences corresponds to the recommendations made for multispectral FreeSurfer segmentation for the hippocampal formation (Iglesias et al. 2015). In particular, the high in-plane resolution 2D-T2 data is meant to help the segmentation by fitting an ex-vivo reference atlas that allows modelling of the molecular layer.

### Hippocampal segmentations

DICOM images were first compressed and then uploaded on a data-sharing system based on the XNAT platform (https://www.xnat.org/, RRID:SCR_003048) that was accessible to all the participating sites. This system automatically anonymizes data during the upload. The downloaded anonymized DICOM images were converted to nifti format using the free dcm2nii tool (http://www.nitrc.org/projects/dcm2nii/, RRID:SCR_014099. Output format: SPM8—3D NIFTI nii). A visual quality control of the acquired images was performed by an expert clinician before the FreeSurfer processing.

A within-session T1 co-registration and averaging was performed to improve the reproducibility of the hippocampal subfield segmentation, as previously described (Marizzoni et al. 2015). From now on, reference to the T1 data always refers to the within-session average of two T1 acquisitions (T1s).

**Fig. 1:**
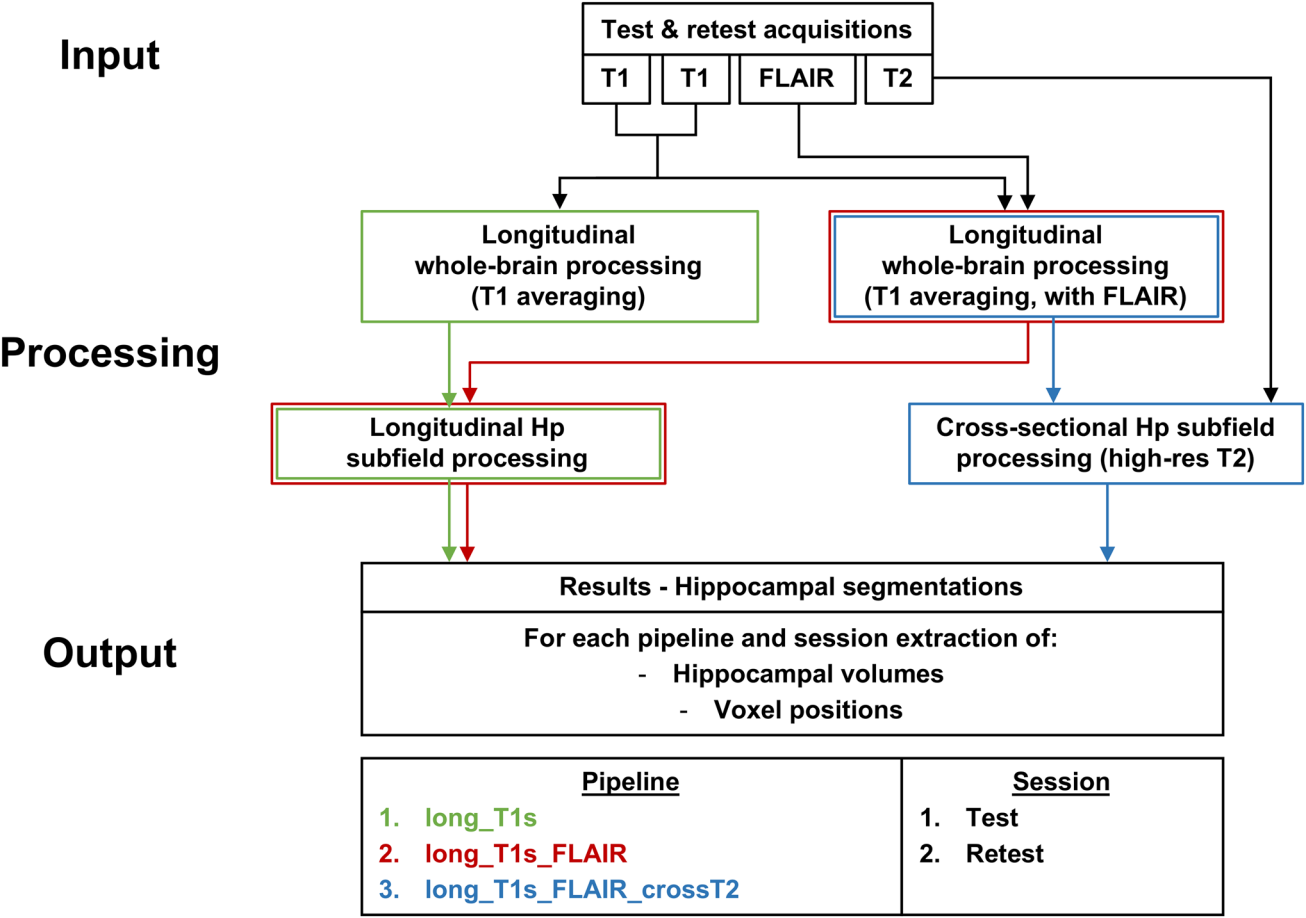
MRI image processing flowchart for the three tested segmentation pipelines (long_T1s, long_T1s_FLAIR and long_T1s_FLAIR_crossT2). *Hp* hippocampus

Figure 1 shows, schematically, the three hippocampal subfield segmentation pipelines implemented in FreeSurfer 6.0. The pipelines differ among themselves in the level of multispectral MRI contrast information used for the subfield segmentations: one contrast (two averaged 3D-T1s; green in Fig. 1), two contrasts (two averaged 3D-T1s and 3D-FLAIR; red in Fig. 1), or three contrasts (two averaged 3D-T1s, 3D-FLAIR, and high-resolution 2D-T2; blue in Fig. 1). The steps of the main analyses are outlined as follows.

Test–retest of the averaged T1 structural images were automatically processed according to the longitudinal pipeline (Reuter et al. 2012) of FreeSurfer 6.0. Specifically, an unbiased within-subject template (Reuter and Fischl 2011) is created using robust, inverse consistent registration (Reuter et al. 2010), and several processing steps are then initialized with common information from the within-subject template. Since FreeSurfer allows the use of FLAIR images to improve the pial surface reconstruction, we tested the longitudinal pipeline both with FLAIR images (red in Fig. 1) and without (green in Fig. 1).

The final step for all the pipelines is the segmentation of the hippocampal subfields. The longitudinal module (Iglesias et al. 2016) was used on both outputs of the FreeSurfer longitudinal pipelines, with only T1s (green in Fig. 1) or T1s and FLAIR (red in Fig. 1). This longitudinal module for the segmentation of the hippocampal subfields does not allow the use of additional high-resolution 2D-T2 images. Therefore, to incorporate the three image contrasts in the hippocampal subfield segmentation, we used the output of the FreeSurfer longitudinal pipeline with 3D-T1 and 3D-FLAIR images, adding the high-resolution 2D-T2 images (blue in Fig. 1) in a cross-sectional approach (Iglesias et al. 2015), where the segmentations at test and retest sessions are done separately.

To abbreviate, we refer to the three hippocampal subfield pipelines with the following labels: long_T1s refers to the full longitudinal pipeline where only two averaged 3D-T1s are used, long_T1s_FLAIR refers to the full longitudinal pipeline where two averaged 3D-T1s and 3D-FLAIR are used, and long_T1s_FLAIR_crossT2 refers to the longitudinal FreeSurfer pipeline with two averaged 3D-T1s and 3D-FLAIR, followed by the cross-sectional pipeline for the segmentation of the hippocampal subfields also using the high-resolution 2D-T2.

FreeSurfer 6.0 hippocampal subfield segmentations included for both brain hemispheres the cornu ammonis areas (CA1, CA2-3, CA4), hippocampal tail (Hp_Tail), subiculum, hippocampal fissure (fissure), presubiculum, parasubiculum, molecular layer (Hp_ML), granule cells in the molecular layer of the dentate gyrus (GC-ML-DG), fimbria, and the hippocampal-amygdala transitional area (HATA). In addition to the whole hippocampus (Whole_Hp, i.e. sum of all hippocampal subfields except hippocampal fissure), we also computed an additional estimate of the whole hippocampus, called Whole_Hp_Fiss, which added the fissure to Whole_HP. No manual edits were performed*.*

On average, it took about 18 h/subject to complete the longitudinal processing that used only T1 images on a Linux workstation (Ubuntu 16.04) equipped with an Intel Xeon E5-1603 v3 CPU (4 × 2.80 GHz processors) and 16 GB of 1866 MHz DDR4 RAM. Adding FLAIR images, the overall computation time increased about 2 h; an additional 1 h was required for the cross-sectional processing with the high-resolution T2 images.

Segmentations were visually examined before the statistical analysis to exclude major errors. A Kruskal–Wallis test was used to evaluate for MRI site effects on the hippocampal volume segmentations. After confirming no significant site effects, the test–retest segmentation data was grouped across sites with a focus on evaluating pipeline effects on test–retest reproducibility metrics.

### Test–retest reproducibility analysis

To assess the test–retest reproducibility of the hippocampal segmentations, we considered for each subject and each segmentation two metrics: the percent absolute reproducibility error (RE) and the DICE coefficient (Van Rijsbergen 1978) across the test–retest sessions.

For each structure, the dimensionless measure RE is the absolute percent difference of the volume with respect to its mean value between test and retest sessions:

$$ {\text{RE}} = 100\, \cdot \,\frac{{\left| {V_{{{\text{retest}}}} - V_{{{\text{test}}}} } \right|}}{{\left( {V_{{{\text{retest}}}} + V_{{{\text{test}}}} } \right)/2}}. $$

The spatial reproducibility was studied by computing the DICE coefficient, which estimated the overlap between the co-registered test–retest volumes of the same hippocampal structure. DICE coefficient is defined as:

$$ {\text{DICE}} = \frac{{\left| {M_{{{\text{retest}}}} \cap M_{{{\text{test}}}} } \right|}}{{\left( {\left| {M_{{{\text{retest}}}} } \right| + \left| {M_{{{\text{test}}}} } \right|} \right)/2}}, $$

where *M*_test_ and *M*_restest_ represent the binary masks of the same hippocampal structure coming from the two different MRI acquisitions. For two identical masks, DICE = 1 if they are identically positioned, whereas DICE value is less than 1 if the spatial overlap is not perfect (reaching zero if there is no overlap at all).

The RE and DICE reproducibility analyses were carried out on the left/right hemisphere average for each segmentation volume. For inter-site analyses, DICE was averaged across the subjects scanned at the same MRI center.

### Statistical analysis

Statistical analysis was performed using R (software version 3.5.1; http://www.r-project.org/, RRID: SCR_001905). A significance level of 5% was adopted in all the analyses.

A Kruskal–Wallis test was used to estimate possible MRI site differences regarding subjects’ ages, genders, test–retest time intervals, and hippocampal volumes. The Kruskal–Wallis test was also used for comparisons of the test–retest reproducibility measures between the three tested FreeSurfer pipelines (long_T1s, long_T1s_FLAIR, and long_T1s_FLAIR_crossT2).

A Wilcoxon test corrected for multiple comparisons (Bonferroni correction) was applied for paired comparisons of the hippocampal volumes and the test–retest reproducibility measures between the three tested FreeSurfer pipelines.

In addition, Bland–Altman plots have been used to visualize the test–retest volume differences of hippocampal structures as a function of mean structure volume for each segmentation pipeline.

## Results

**Fig. 2:**
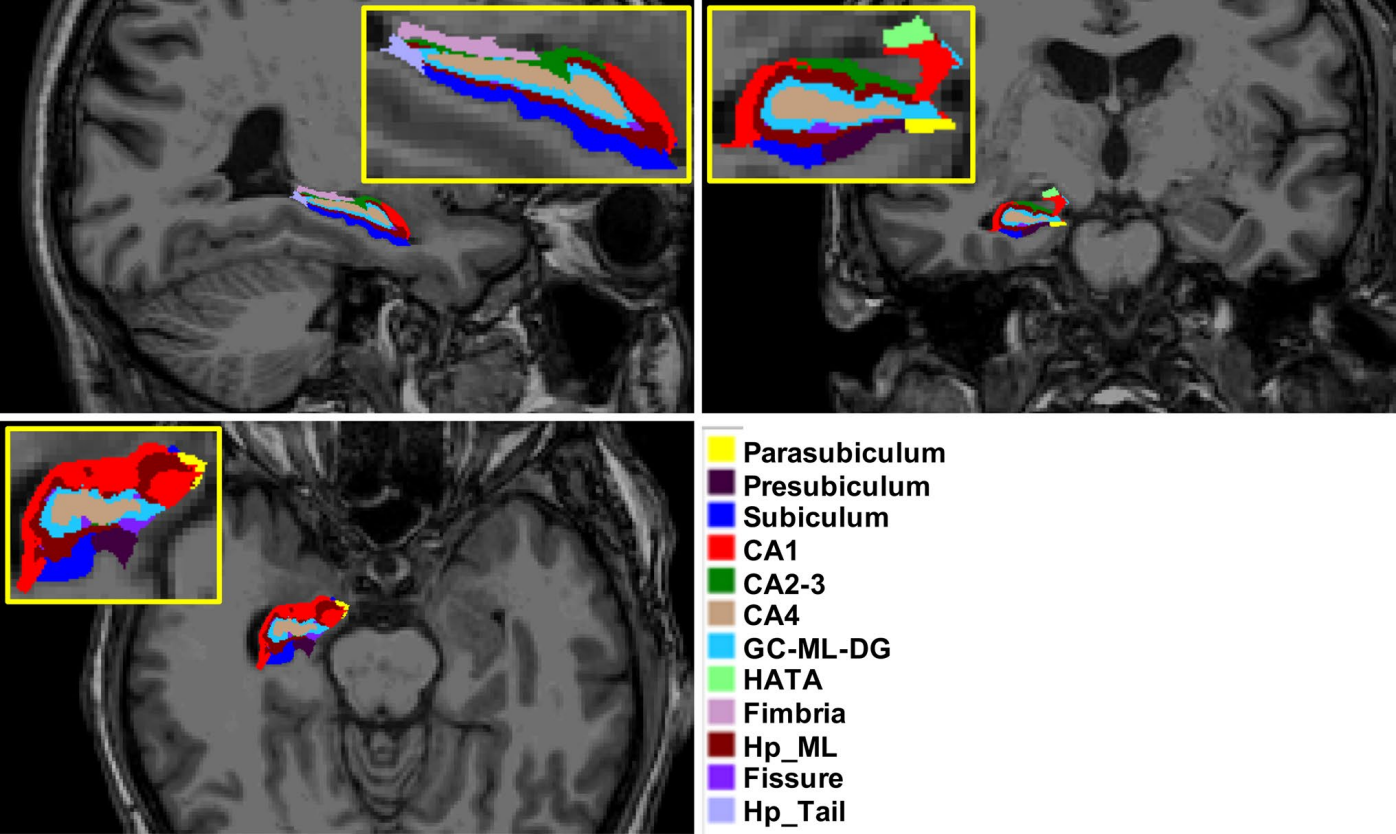
Illustration of the hippocampal subfield segmentations in sagittal (top left), axial (bottom), and coronal (top right) views. Subfield structures of a sample subject (site 1, session 1, longitudinal pipeline with FLAIR) are overlaid on the corresponding averaged T1-weighted image generated by FreeSurfer 6.0 during the automated processing. The images were made using the FreeView visualization tool (https://surfer.nmr.mgh.harvard.edu/fswiki/FreeviewGuide/). *CA1, CA2-3, CA4* cornu ammonis areas, *GC-ML-DG* granule cells in the molecular layer of the dentate gyrus, *HATA* hippocampal-amygdala transitional area, *Hp_ML* molecular layer, *Fissure* hippocampal fissure, *Hp_Tail* hippocampal tail

No major segmentation errors were found upon visual inspection. Figure 2 shows hippocampal segmentation results overlaid on the corresponding T1 from a sample subject.

A Kruskal–Wallis test confirmed that there were no statistically significant differences across MRI sites regarding subject’s age (*p* = 0.054), gender (*p* = 0.71), and test–retest time interval (*p* = 0.14). Further, hippocampal volume showed no MRI site effects, regardless of segmentation pipeline (*p* = 0.13–0.21).

**Fig. 3:**
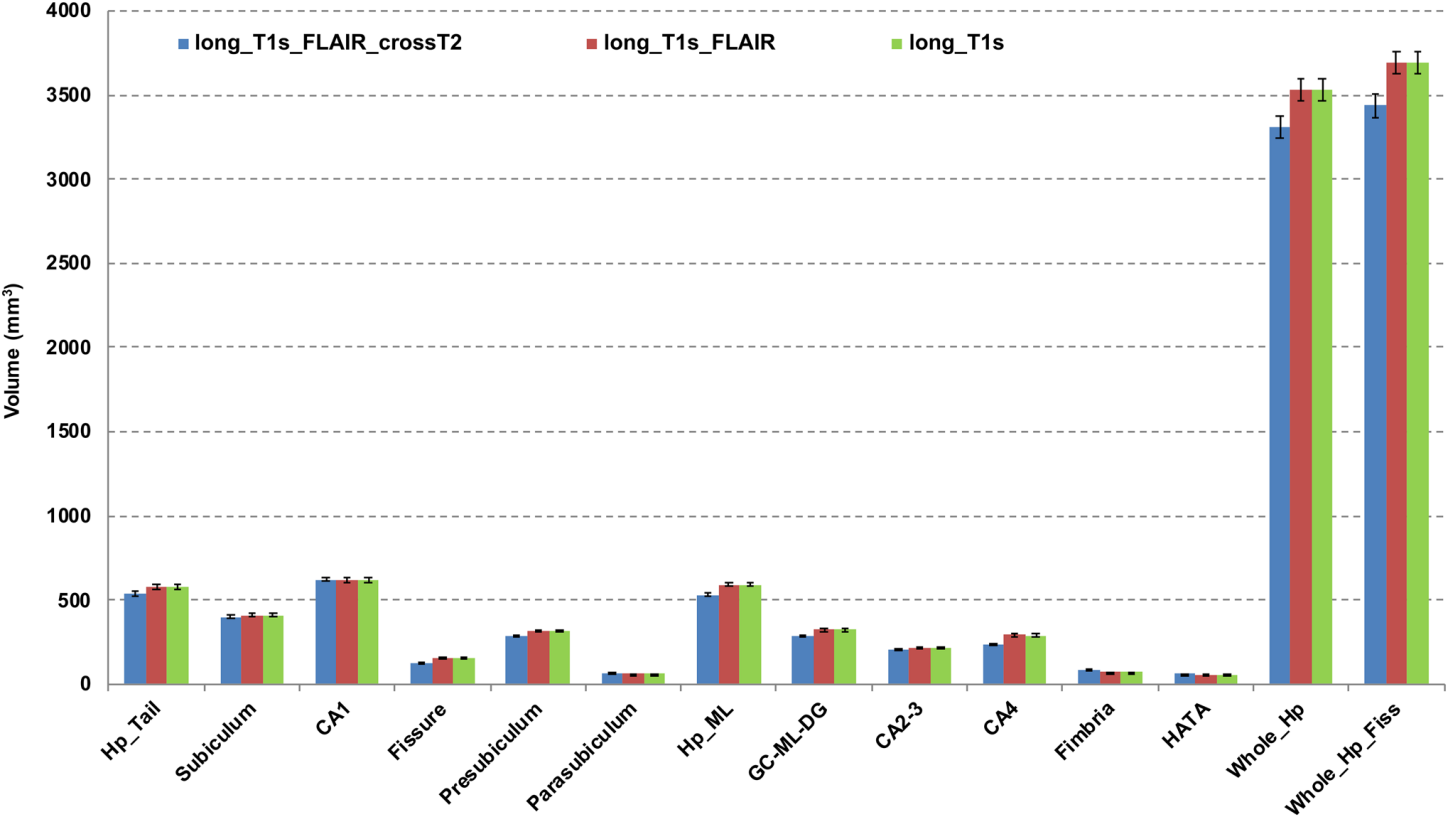
Volume estimates of whole hippocampus and its subfields for the three tested segmentation pipelines (long_T1s, long_T1s_FLAIR and long_T1s_FLAIR_crossT2). The last region on the right refers to the structure obtained merging the whole hippocampus and fissure. Error bars represent the standard deviation from the mean. *CA1, CA2-3, CA4* cornu ammonis areas, *GC-ML-DG* granule cells in the molecular layer of the dentate gyrus, *HATA* hippocampal-amygdala transitional area, *Hp_ML* molecular layer, *Fissure* hippocampal fissure, *Hp_Tail* hippocampal tail

A statistically significant difference was found (Wilcoxon test, *p* < 0.001) between the hippocampal volume segmented using the longitudinal pipelines (mean hippocampal volume for both the longitudinal pipelines: 3530 ± 70 mm^3^) in comparison to the cross-sectional one (mean hippocampal volume: 3310 ± 10 mm^3^). Figure 3 shows the distribution of hippocampal subfield volumes for the three pipelines.

To summarize the data, unless otherwise stated, in what follows we grouped the data across sites, averaging right and left hemispheres for each hippocampal structure.

## Test–retest reproducibility of whole hippocampus and its subfields with long_T1s_FLAIR pipeline

In this section we focus on the reproducibility results from the long_T1s_FLAIR pipeline, first reporting the test–retest RE and then the DICE coefficients for spatial overlap. The comparison across pipelines is reported in the following subsection.

**Fig. 4:**
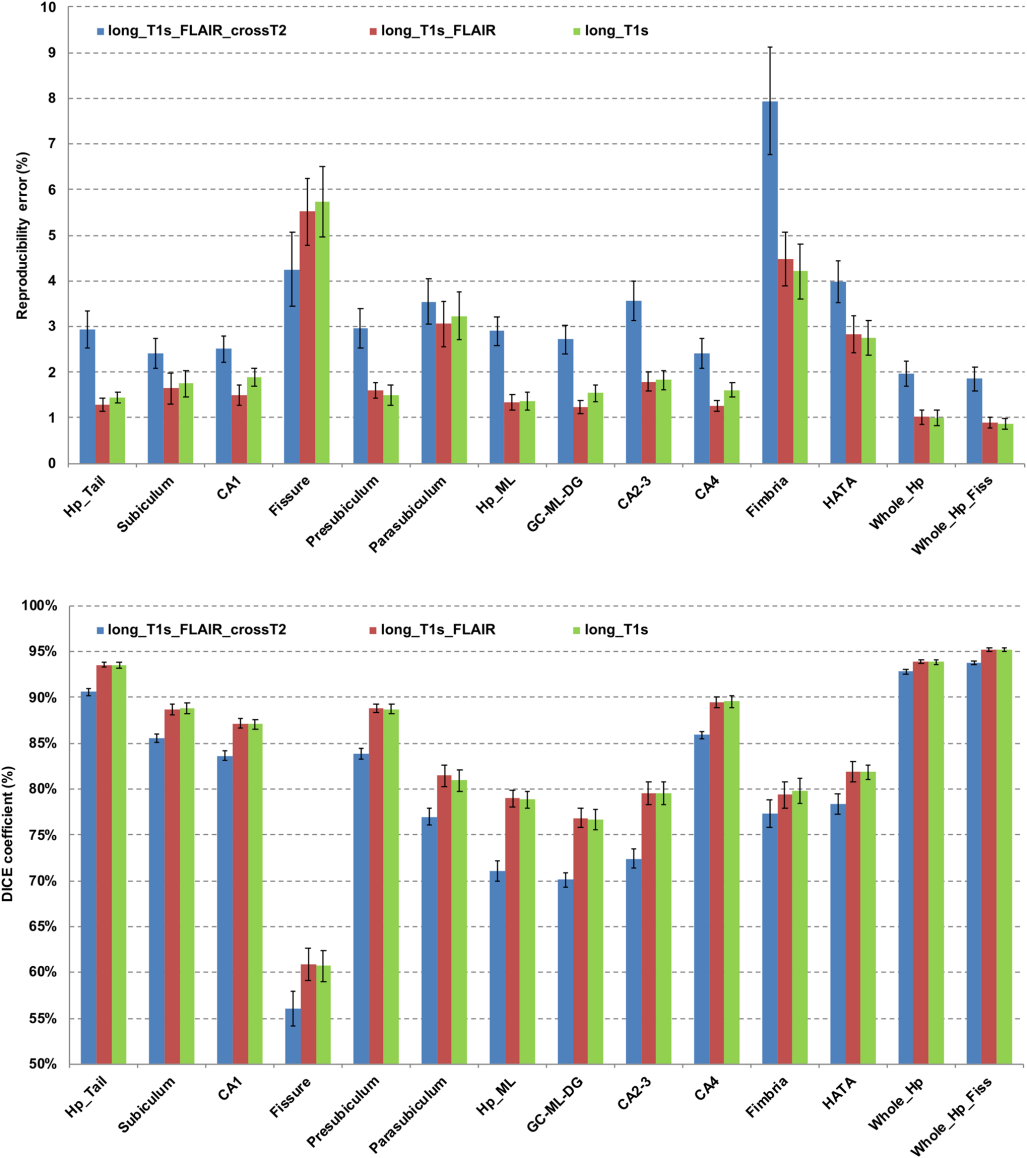
Test–retest volume reproducibility error (upper panel) and spatial reproducibility (DICE coefficient, lower panel) of whole hippocampus and its subfields for the three tested segmentation pipelines (long_T1s, long_T1s_FLAIR and long_T1s_FLAIR_crossT2). The last region on the right is relative to the structure obtained merging the whole hippocampus and fissure. Error bars represent the standard deviation from the mean. *CA1, CA2-3, CA4* cornu ammonis areas, *GC-ML-DG* granule cells in the molecular layer of the dentate gyrus, *HATA* hippocampal-amygdala transitional area, *Hp_ML* molecular layer, *Fissure* hippocampal fissure, *Hp_Tail* hippocampal tail

Figure 4 (upper panel) shows the test–retest RE (average and standard deviation of RE across the whole group) for each hippocampal subfield segmentation (left and right hemisphere averages) separately for the three pipelines.

RE varies across hippocampal structures from 1 to 6% (Fig. 4, upper panel, red bars). In particular, mean test–retest RE among MRI sites was ≈1% and ≈0.9% for Whole_Hp and Whole_Hp_Fiss, respectively. With regard to the hippocampal subfields, the mean test–retest RE was ≈6% for fissure, < 5% for fimbria, ≈3% for parasubiculum, < 3% for HATA, and < 2% for the other structures.

The DICE coefficients of spatial overlap can be seen in the lower panel of Fig. 4 (red bars, long_T1s_FLAIR pipeline). The spatial reproducibility (DICE) was ≈ 95.2% for Whole_Hp_Fiss, ≈93.9% for Whole_Hp, ≈ 93.5% for Hp_Tail, > 85% for subiculum, presubiculum, CA1, and CA4, > 80% for parasubiculum and HATA, > 75% for GC-ML-DG, Hp_ML, CA2-3, and fimbria, ≈61% for the fissure.

**Fig. 5:**
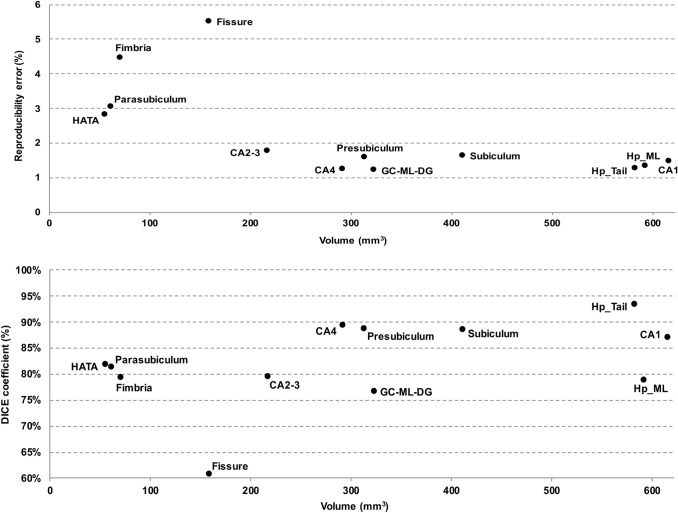
Test–retest volume reproducibility error (upper panel) and spatial reproducibility (DICE coefficient, lower panel) of hippocampal subfields obtained from the long_T1s_FLAIR pipeline as a function of the structure volume. The volumes were averaged across hemispheres, sessions, and subjects. *CA1, CA2-3, CA4* cornu ammonis areas, *GC-ML-DG* granule cells in the molecular layer of the dentate gyrus, *HATA* hippocampal-amygdala transitional area, *Hp_ML* molecular layer, *Fissure* hippocampal fissure, *Hp_Tail* hippocampal tail

Figure 5 shows the distribution of test–retest reproducibility metrics as functions of hippocampal structure volume. The RE distribution (Fig. 5, top), shows a fairly stable reproducibility in the range of 1–2% for structures larger than 200 mm^3^, with reproducibility loss for smaller structures (fissure, fimbria, parasubiculum, HATA). The DICE coefficient distribution (Fig. 5, bottom), shows an overall good spatial reproducibility (75–95%) across volumes except for the fissure.

### Segmentation reproducibility: comparison across pipelines

A Kruskal–Wallis test showed a significant difference of the reproducibility metrics across the three pipelines, both for RE (*p* < 0.001) and DICE coefficient (*p* < 0.001).

The post hoc Wilcoxon test showed a significant decrease of hippocampal structures reproducibility in the long_T1s_FLAIR_crossT2 pipeline with respect to the other two longitudinal pipelines, both in terms of RE and DICE coefficient (*p* < 0.001 in all cases). The higher reproducibility of the longitudinal pipelines can be seen in Fig. 4 (upper panel). Except for the hippocampal fissure, there was an average reduction of 0.4–3.6% in the test–retest RE of all segmentations. Similarly (Fig. 4, lower panel), with regard to the reproducibility of spatial overlap of the segmentations, the longitudinal pipelines showed a higher spatial reproducibility (1.1–7.8% of DICE improvement) in all hippocampal structures relative to the long_T1s_FLAIR_crossT2 pipeline.

Moreover, when comparing the two longitudinal pipelines, long_T1s_FLAIR provided a small but significant reproducibility improvement in terms of test–retest RE (Wilcoxon test, *p* = 0.015). Meanwhile, no significant DICE differences were found between the two longitudinal pipelines (Wilcoxon test).

Bland–Altman plots (Suppl. Figure 1) show the test–retest volume differences of hippocampal structures as a function of mean structure volume for each segmentation pipeline. The longitudinal pipelines (long_T1s, long_T1s_FLAIR) substantially reduced volume differences across all subregions, except for the fissure, with respect to long_T1s_FLAIR_crossT2. For some structures (e.g. CA1, CA4, GC-ML-DG, parasubiculum), long_T1s_FLAIR slightly reduced volume differences with respect to long_T1s.

Summarizing, the two longitudinal pipelines (long_T1s and long_T1s_FLAIR) give more reproducible hippocampal segmentations than the cross-sectional pipeline (long_T1s_FLAIR_crossT2), with a marginal but significant reproducibility improvement for the long_T1s_FLAIR pipeline.

## Discussion

It is currently unclear how the choice of different anatomical MRI input data (T1, FLAIR, high-resolution T2) and choice of FreeSurfer segmentation pipelines affect the reproducibility of hippocampal subfield segmentations. In this multicentric study, we evaluated the test–retest reproducibility of automated hippocampal subfield segmentations using three different FreeSurfer 6.0 pipelines in 22 healthy subjects scanned twice using multispectral acquisitions in four clinical 3T MRI centers. To the best of our knowledge, we provide for the first time a quantitative characterization of hippocampal subfield segmentation multicentric reproducibility using 3D-FLAIR and high-resolution 2D-T2 input images in addition to standard 3D-T1 images. The most important findings of this study are two: (1) the longitudinal hippocampal subfield segmentation pipelines are superior to the cross-sectional one using the high-resolution 2D-T2 data, (2) use of 3D-T1s and 3D-FLAIR in the longitudinal pipelines offers marginal but significant reproducibility improvements relative to the use of only 3D-T1 data.

The hippocampal formation is a brain region that is affected by several neurological and psychiatric disorders and its atrophy is already used to help enrich recruitment into AD clinical trials, as reported by the European Medicines Agency in EMA/CHMP/SAWP/809208/2011. Morphometric information about its sub-regions could contribute to a differential diagnosis in pathological states. For example, Iglesias et al. (2015) showed that the discrimination of mildly cognitive impaired and mild AD patients improved using volumetric data of hippocampal subfields relative to the whole hippocampus.

In our study, we found that the longitudinal pipeline for hippocampal subfield segmentation (Iglesias et al. 2016) gave an overall higher test–retest reproducibility (percent volume errors in the range of 1–6% across structures) compared to the cross-sectional pipeline (Iglesias et al. 2015) with high-resolution 2D-T2 images (2–8% across structures). The improved performance of the longitudinal pipelines with respect to the cross-sectional one is in good agreement with other 3T studies, even if they used only 3D-T1 and older versions of FreeSurfer, which found similar results in the whole hippocampus (Jovicich et al. 2013) and with the hippocampal subfields (Worker et al. 2018). In addition, the high-resolution 2D-T2 images are expected to have higher operator-dependent variability across sessions since they cover only a part of the brain and need to be oriented perpendicular to the hippocampus by the operator each time. Instead, the 3D-T1 and 3D-FLAIR volumes, being full-brain, are less operator dependent. Further, using the three image contrasts needed for the long_T1s_FLAIR_crossT2 pipeline also requires a slightly longer acquisition time, which results in higher sensitivity to head motion effects that may affect the segmentation (Iglesias et al. 2015) and therefore its reproducibility. The higher REs reported by Worker et al. (2018) are most likely related to a combination of acquisition factors, in particular the use of only one 3D-T1 volume per subject. The hippocampal subfield reproducibility findings of our long_T1s pipeline are consistent with the study from Marizzoni et al. (2015), which also used two averaged 3D-T1s but an older version of FreeSurfer. Both studies showed that reproducibility and DICE coefficients get worse for structures smaller than 200 mm^3^ (such as the hippocampal fissure and fimbria). For structures with larger volumes, the RE is about 1–2% and DICE in the range of 0.75–0.95. Adding a 3D-FLAIR volume for the pial surface reconstruction in the longitudinal pipeline resulted in a small but statistically significant improvement of test–retest RE.

In agreement with previous studies (Van Leemput et al. 2009; Marizzoni et al. 2015), test–retest RE were higher for the smaller volumes, probably because partial volume effects influence the segmentation of smaller regions in a greater percentage. A possible improvement can be achieved by merging close sub-volumes (Mueller et al. 2018). For example, we found an improvement in the test–retest reproducibility of the whole hippocampus by merging it with fissure, which was the least stable subfield. The boundary between hippocampal fissure (i.e. the vestigial space located between the molecular layer and the dentate gyrus) and the external cerebrospinal fluid may contribute to the lower test–retest reproducibility of this structure. In addition, its shape and small size may make this region more susceptible to partial volume effects, compromising the closer structures as well as the whole hippocampus.

Manual delineation represents the gold standard for brain structure segmentation from MRI images. However, for large datasets it is highly time-consuming and requires a very specific expertise. To the best of our knowledge, only one study (de Flores et al. 2015), has so far compared manual and automatic segmentations of the hippocampal subfields (using the previous 5.1 FreeSurfer version). Thus, future accuracy validations of the FreeSurfer 6.0 algorithm would be useful. In particular, previous studies suggest that the use of high-resolution T2 MRI data offers higher segmentation accuracy for hippocampal subfields (Mueller et al. 2018). Therefore, the extension of the longitudinal FreeSurfer pipeline to the use of such type of MRI data seems promising.

This study has several limitations, which briefly include the following experimental issues: sample size, MR scanner bias, reproducibility assessments without accuracy estimations, no evaluation of disease effects, only one automated segmentation method. Our sample size was small (22 subjects) and corresponded to only two repeated measures from four MRI sites having a bias towards Philips scanners (3 out of 4). Our analysis was limited to the evaluation of reproducibility, while the accuracy of segmented regions was not assessed. Such studies would help clarify the bias towards larger hippocampal volumes that we observed with the longitudinal pipelines in comparison to the cross-sectional pipeline using high-resolution 2D-T2 data. With regard to segmentation accuracy, future studies are needed to further validate FreeSurfer and other segmentation methods against the gold standard manual segmentation, potentially showing the advantages of multispectral contrasts in the segmentation of hippocampal subfields. Another limitation is that, being limited to the reproducibility of healthy subjects, we did not assess the sensitivity that the different segmentation pipelines have to detect disease-related changes. Our imaging consortium is currently completing the acquisition of a longitudinal cohort of mildly cognitively impaired subjects. Such data will allow us to evaluate the sensitivity of different pipelines to track disease progression in future studies. Other public datasets with disease samples including multispectral anatomical data may also be considered. Lastly, our study was limited to the evaluation of automated segmentation pipelines from FreeSurfer. Other segmentation tools exist but are beyond the scope of this study (Yushkevich et al. 2015a).

A more general open challenge in the field remains the harmonization across various different hippocampal subfield segmentation protocols that are available and continuously improved (Yushkevich et al. 2015b; Wisse et al. 2017; Xie et al. 2018). A distributed public effort enabling access to multispectral MRI data, access to manually edited segmentations, or even the possibility to contribute by manually editing segmentations, may help provide a common reference dataset against which to compare and improve new segmentation protocols.

## Conclusions

This is the first study that compares the reproducibility of hippocampal subfield segmentations (FreeSurfer 6.0) derived from single and multi-spectral structural MRI data. The segmentation pipelines used the average of two within-session 3D-T1s, either alone or with a 3D-FLAIR using the longitudinal stream. A third pipeline included, in addition to the averaged T1 and FLAIR, a high-resolution 2D-T2 in the cross-sectional stream.

We showed that the choice of automated segmentation pipeline and choice of multispectral structural MRI data used in the segmentation can significantly affect both the volumes and the test–retest reproducibility of human brain hippocampal subfield volumes as measured by FreeSurfer 6.0 in a 3T multicentric study. We found that the longitudinal pipeline using two 3D-T1s and a 3D-FLAIR gave the highest reproducibility relative to the use of a longitudinal pipeline with only two 3D-T1s or a cross-sectional pipeline using two 3D-T1s, a 3D-FLAIR, and a high-resolution 2D-T2. Importantly, the segmentation of most hippocampal subfields was possible with no reproducibility costs relative to the segmentation of the whole hippocampus. Our results support the use of FreeSurfer automated segmentation of hippocampal subfields in clinical studies to develop new biomarkers for diagnosis, staging, progression, and evaluation of treatment response in neuropsychiatric diseases. The extension of the longitudinal pipeline with the use of high-resolution T2 data might offer further reproducibility improvements which should also be evaluated in terms of segmentation accuracy.

## Electronic supplementary material

Below is the link to the electronic supplementary material.

**Suppl. Fig. 1** Bland-Altman plots showing test-retest volume differences ofhippocampal structures as a function of mean structure volume for each segmentationpipeline (long_T1s, long_T1s_FLAIR and long_T1s_FLAIR_crossT2). For eachstructure and pipeline, the plot shows the test-retest structure mean volume and testretestdifference for each subject (left hemisphere: circles, right hemisphere: plus sign),with the mean volume difference and the 95% confidence intervals (solid lines) (PDF 1184 KB)
