# Supplementary figures and images for: Automatic multispectral MRI segmentation of human hippocampal subfields: an evaluation of multicentric test–retest reproducibility
Source: Brain Struct Funct. 2020 Nov 24;226(1):137–50. doi: 10.1007/s00429-020-02172-w (PMC7817563; doi:10.1007/s00429-020-02172-w)

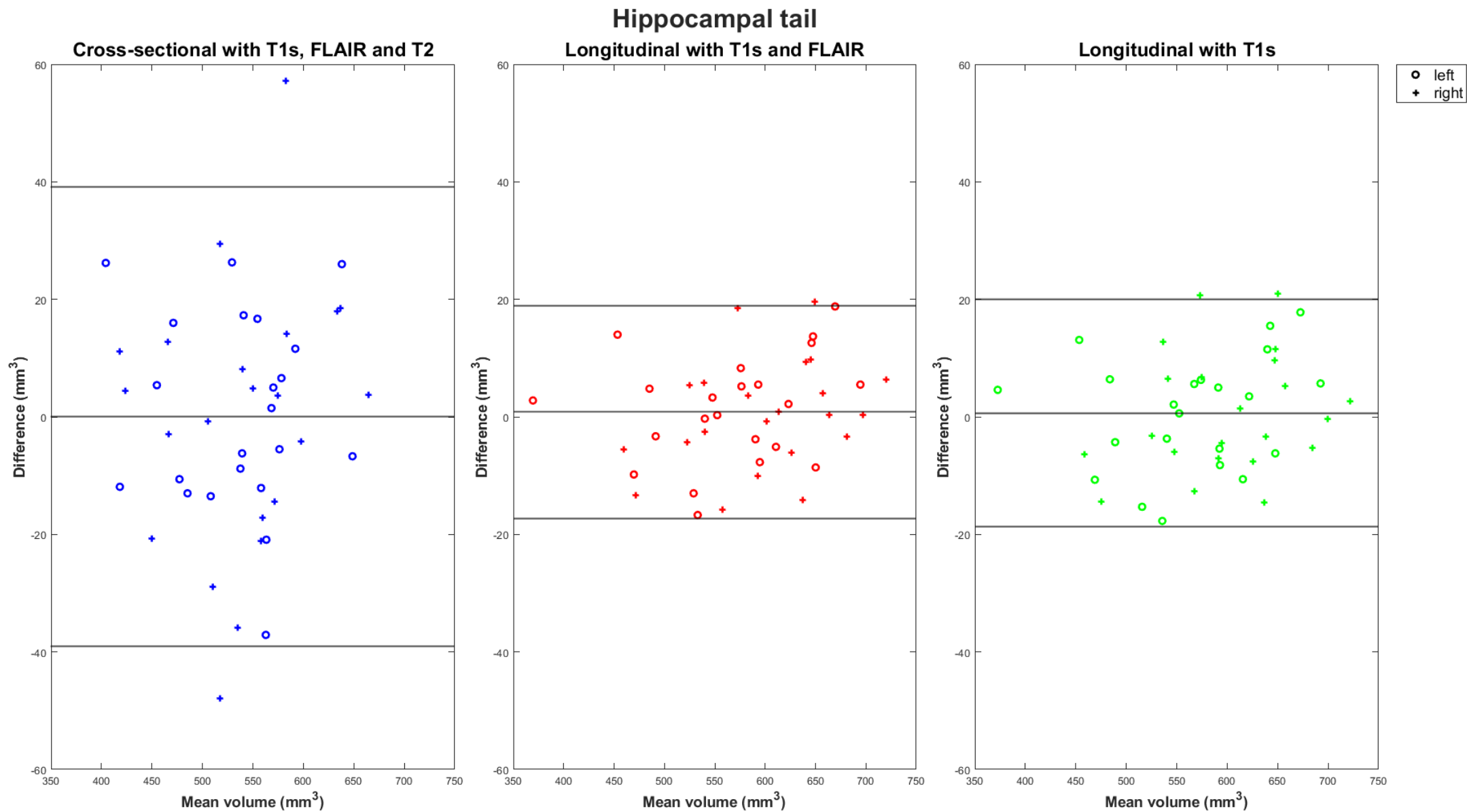

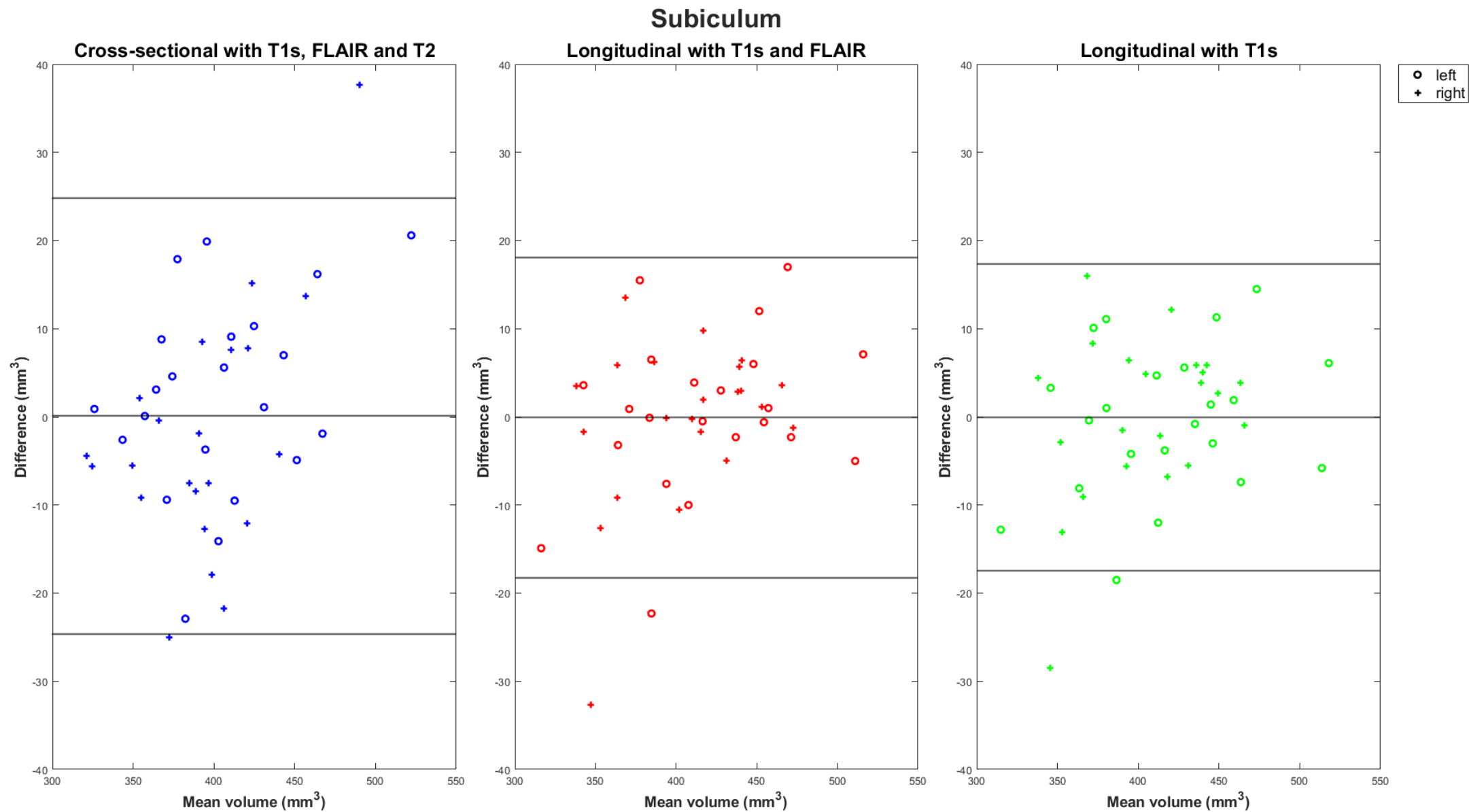

## CA1

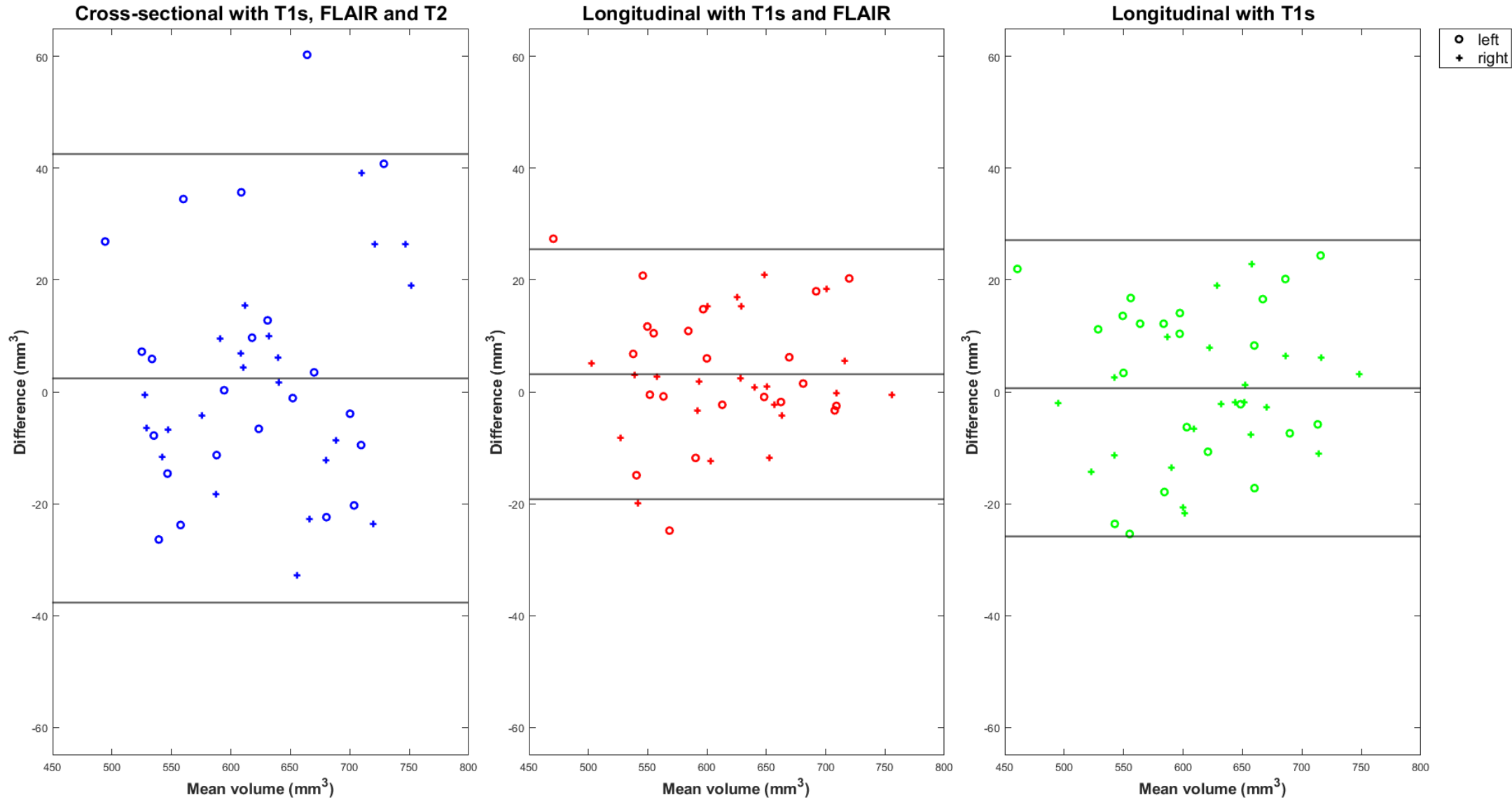

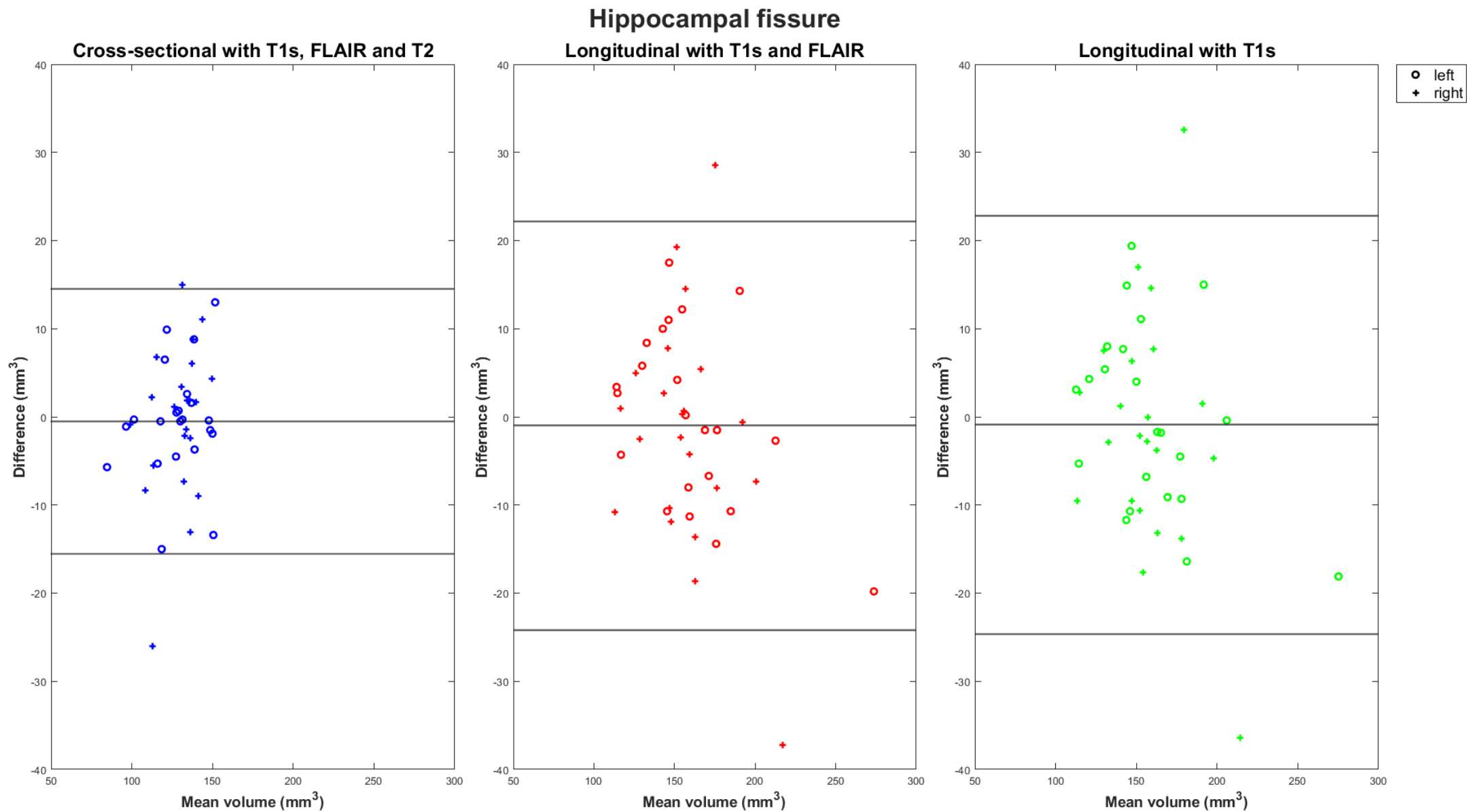

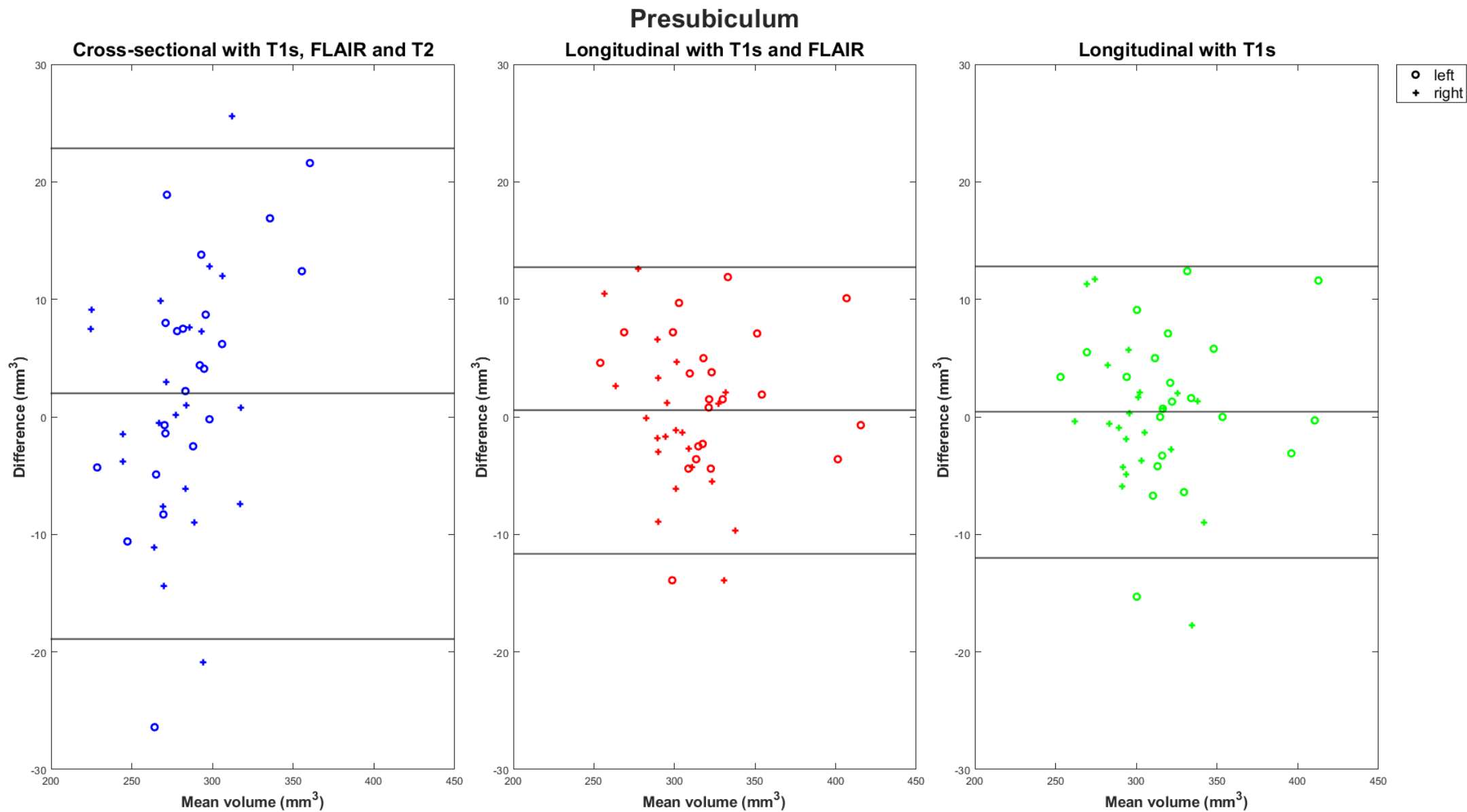

## Parasubiculum

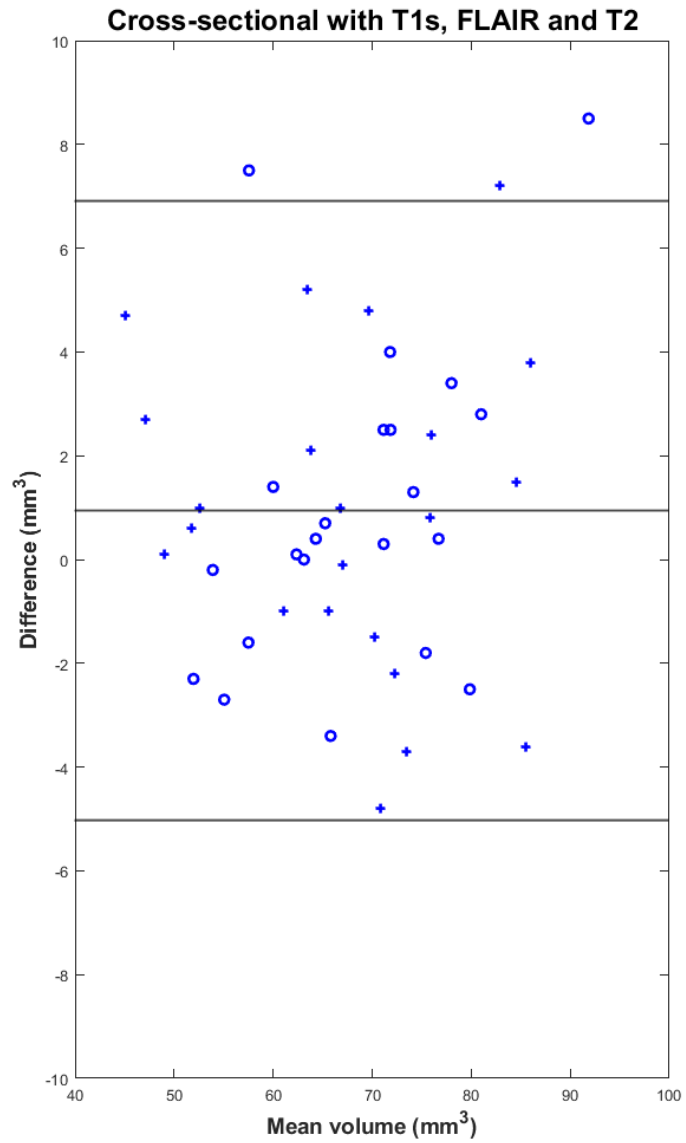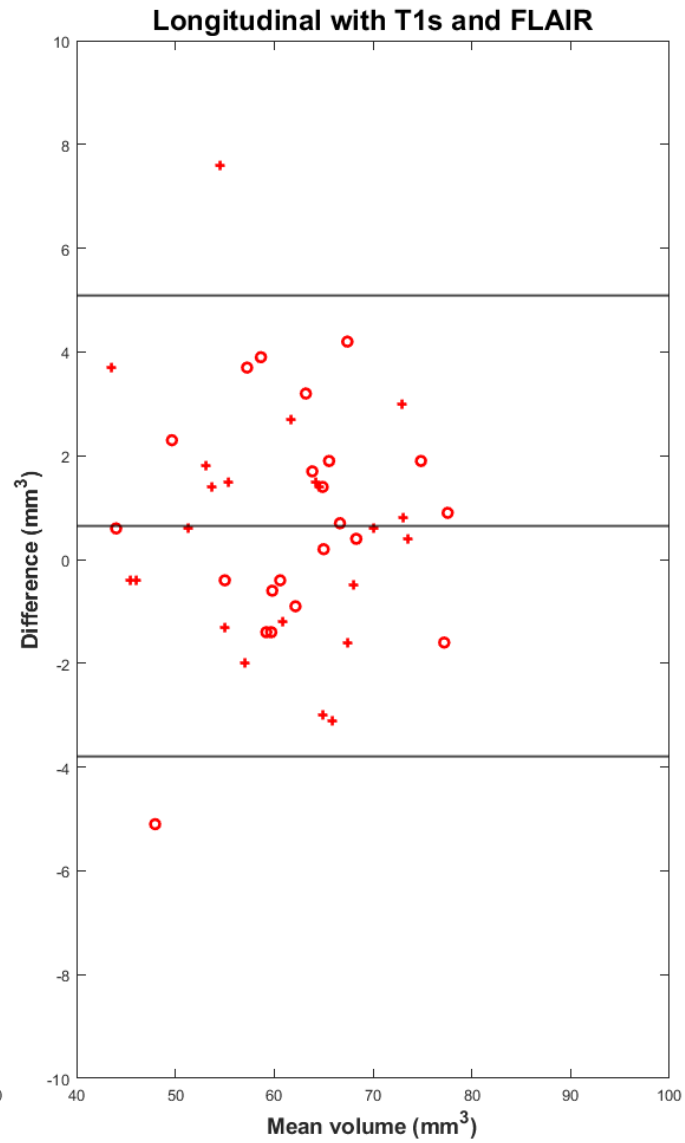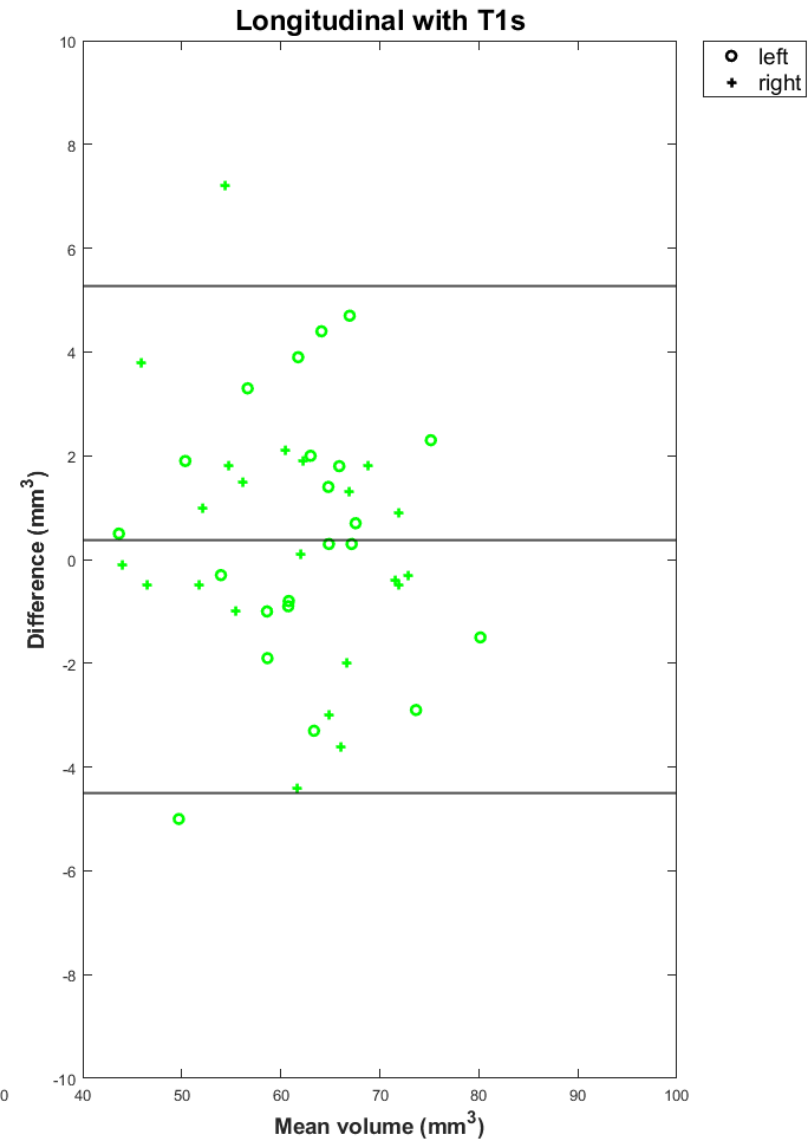

## Molecular layer

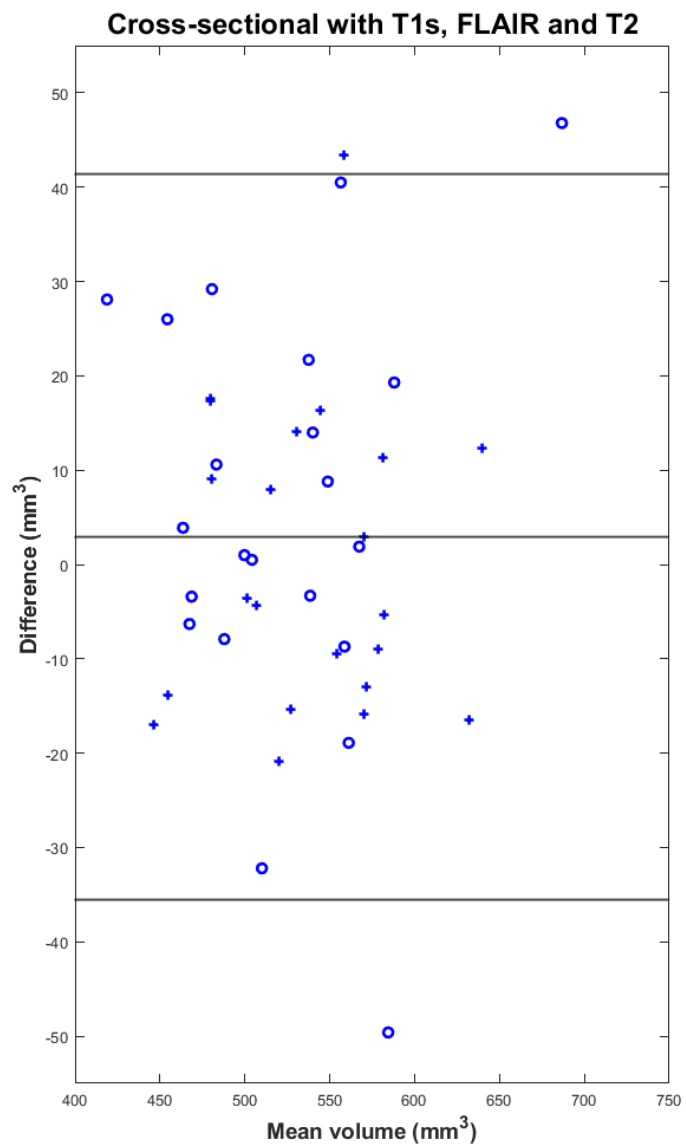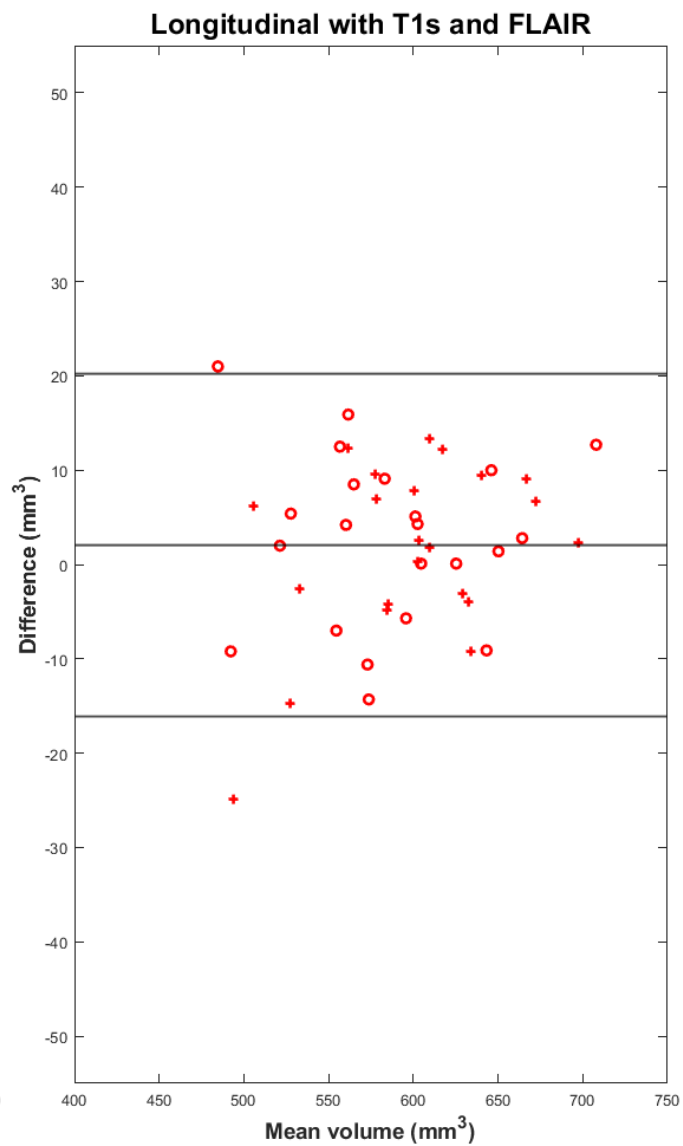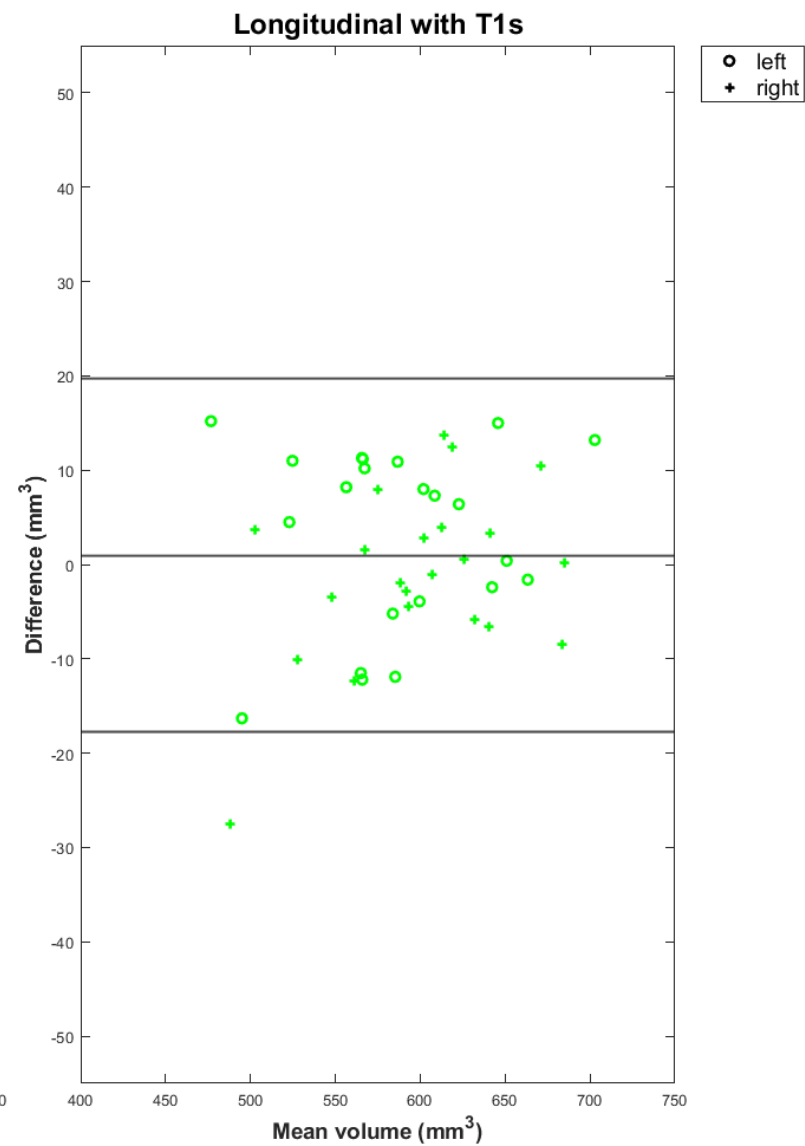

## GC-ML-DG

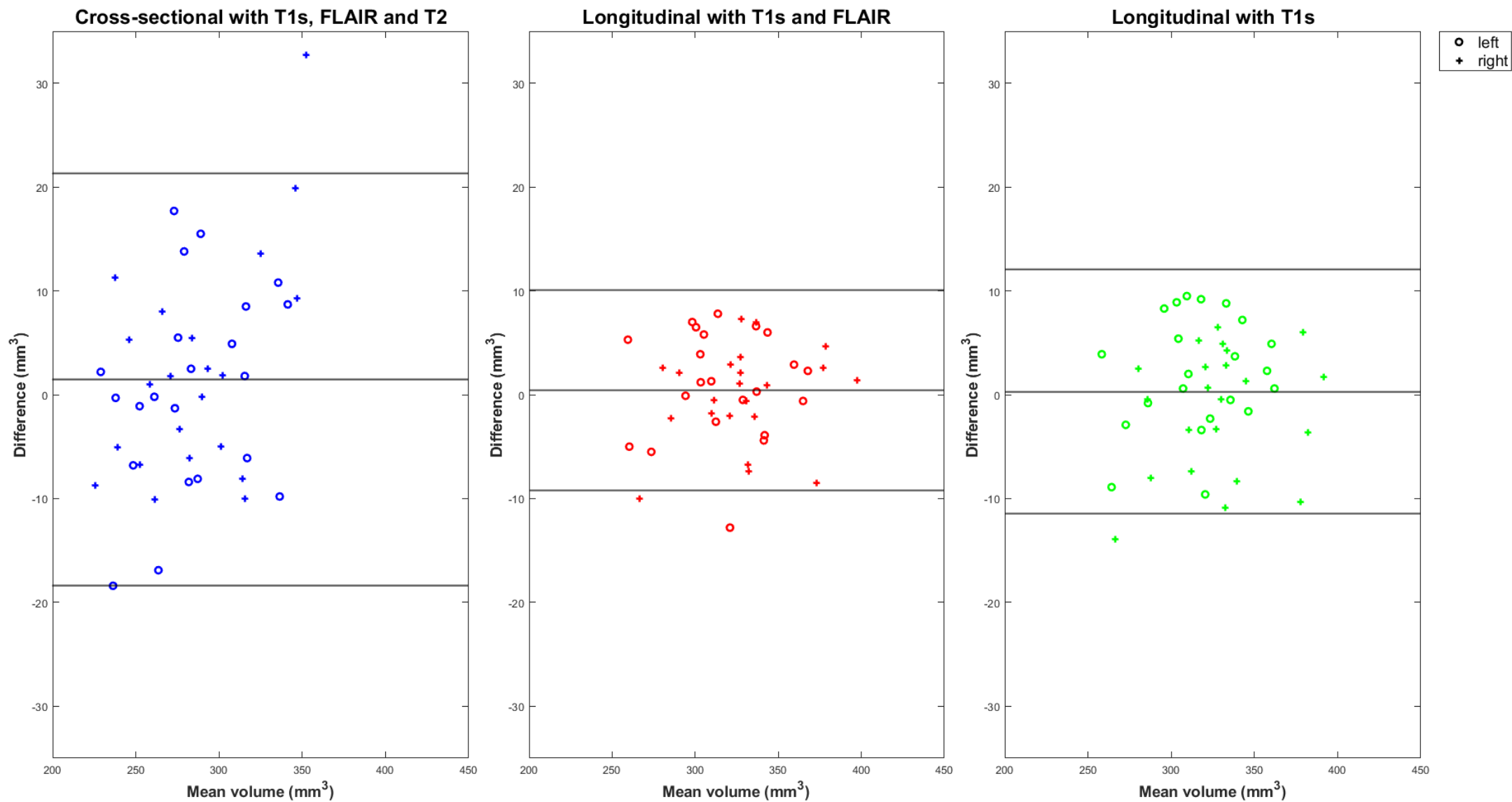

## CA2-3

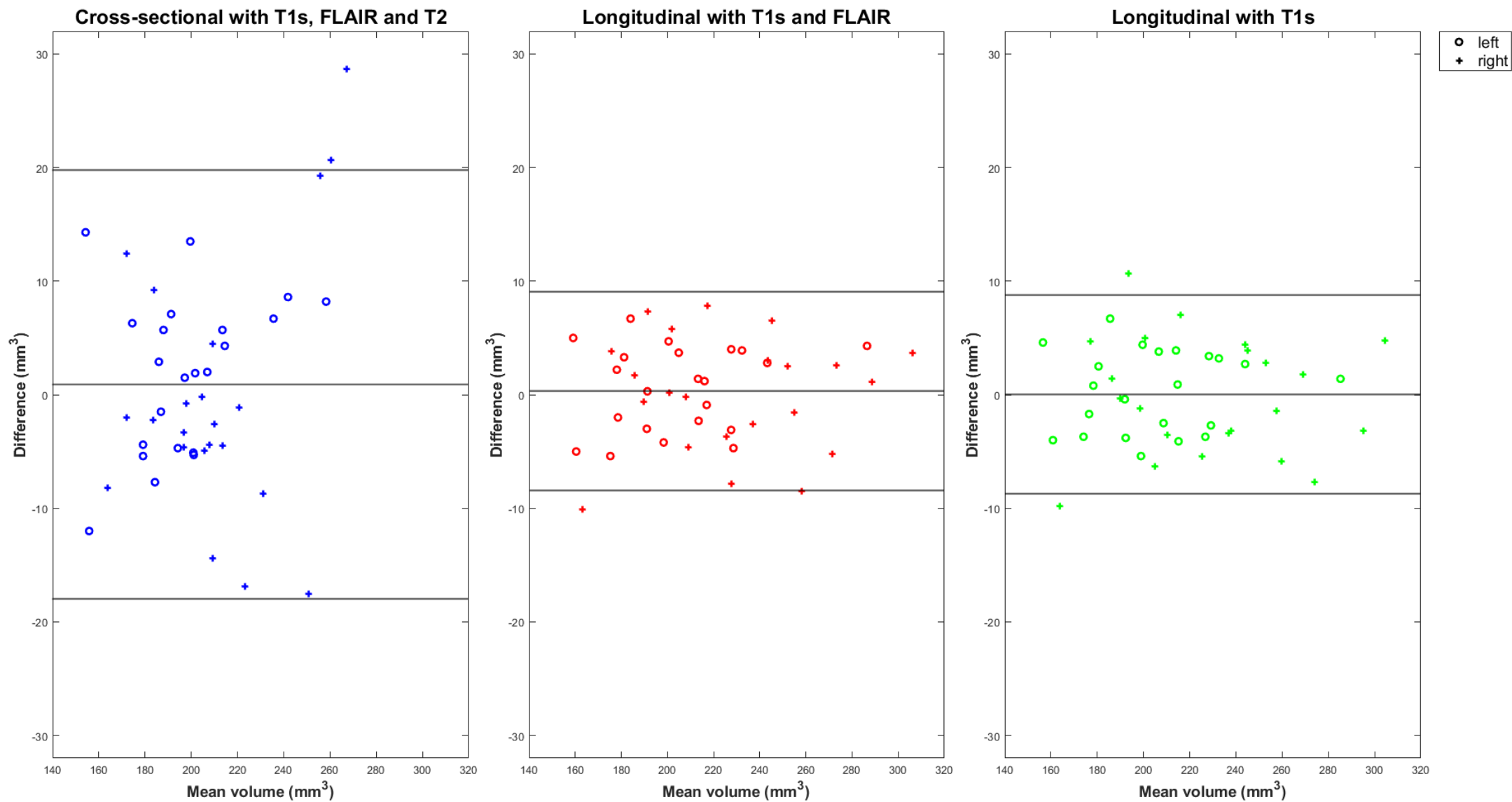

## CA4

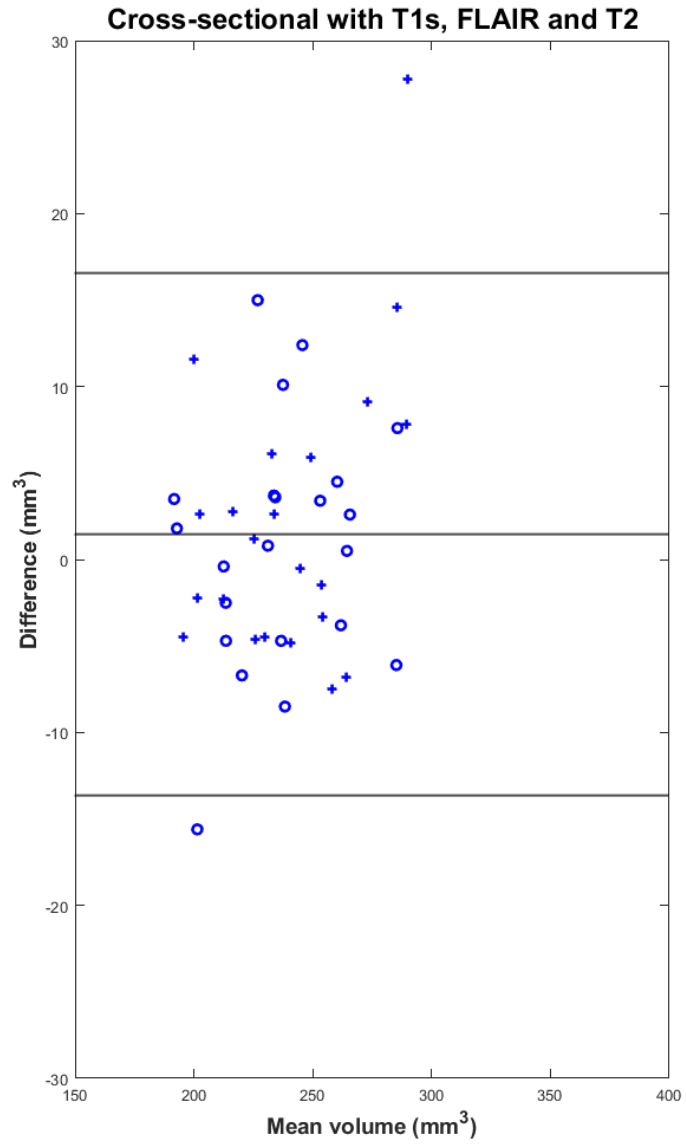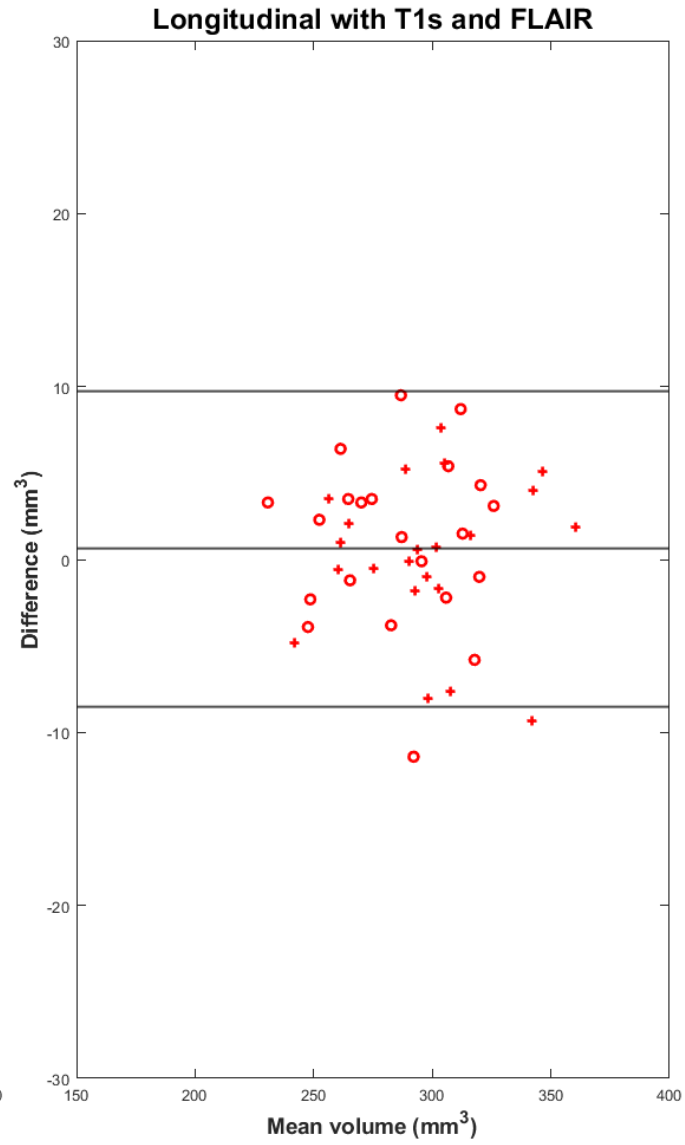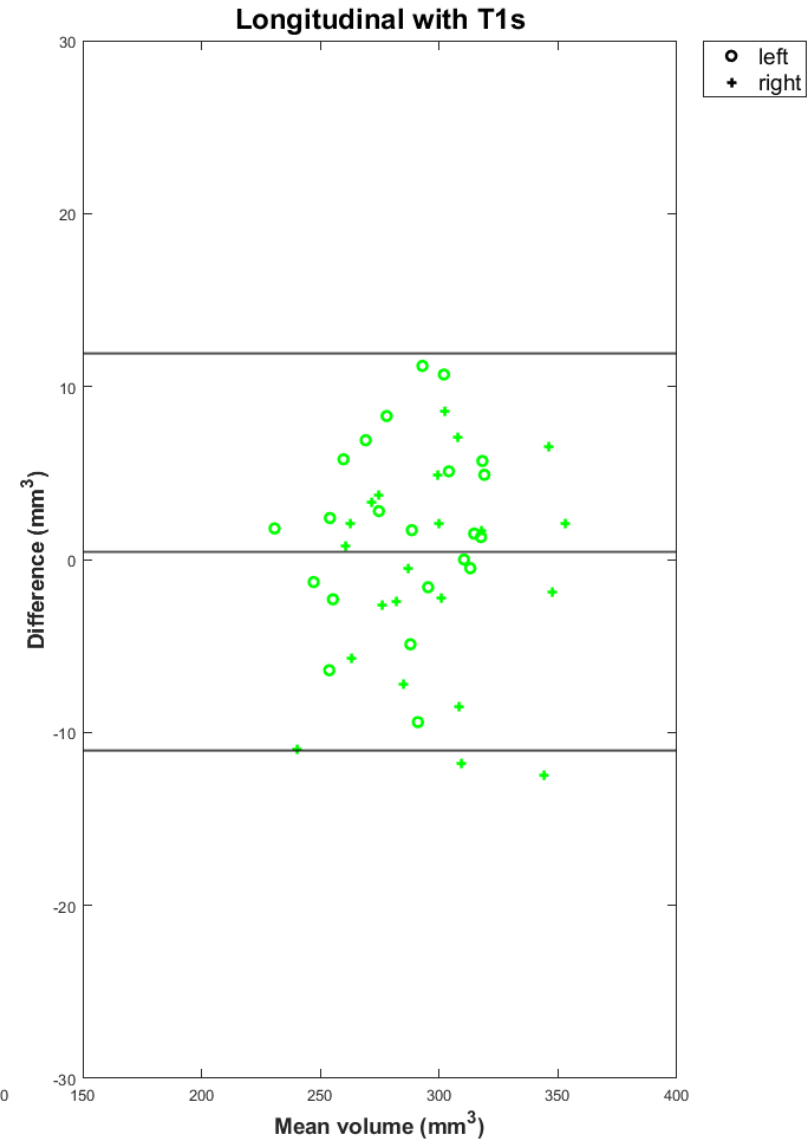

## Fimbria

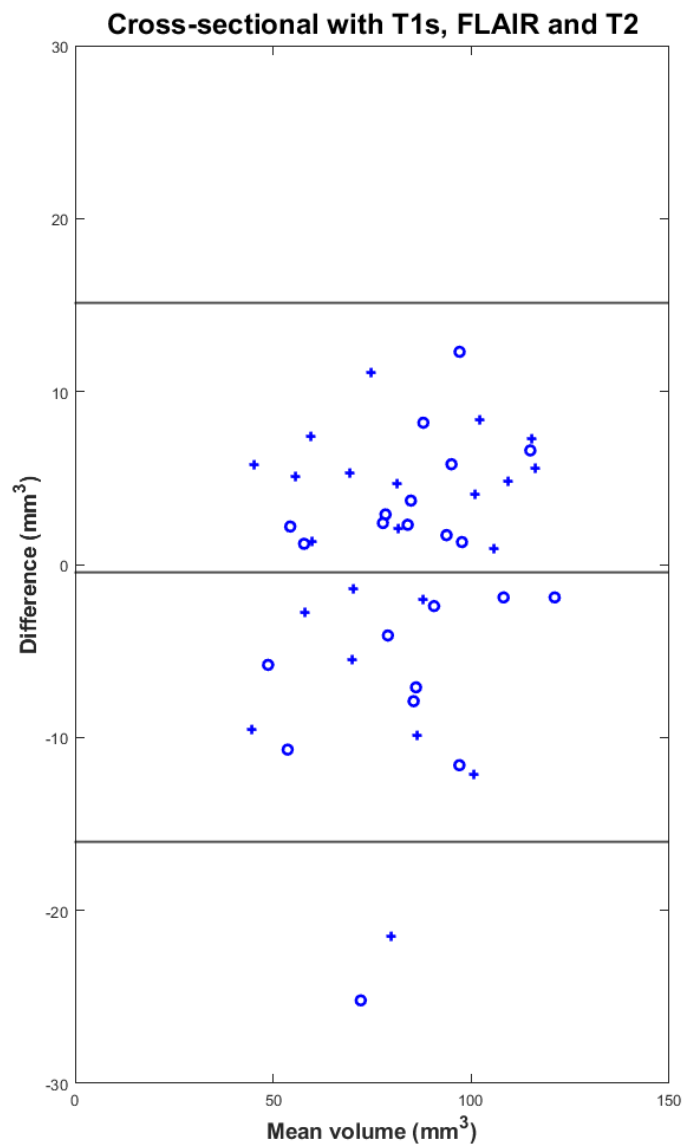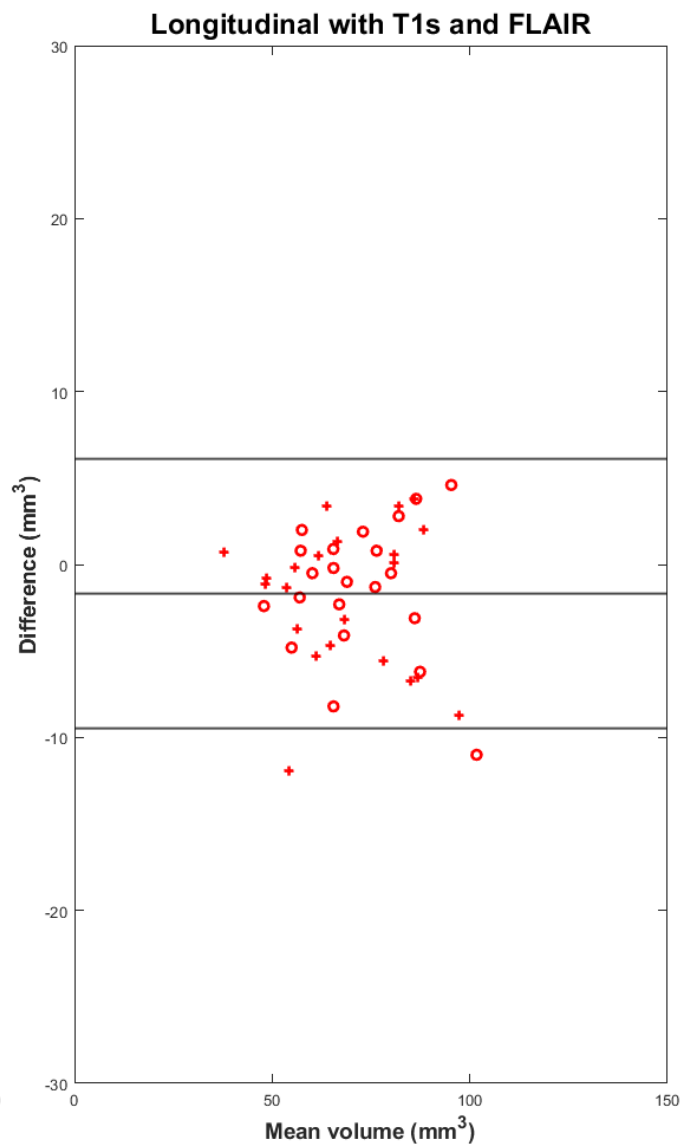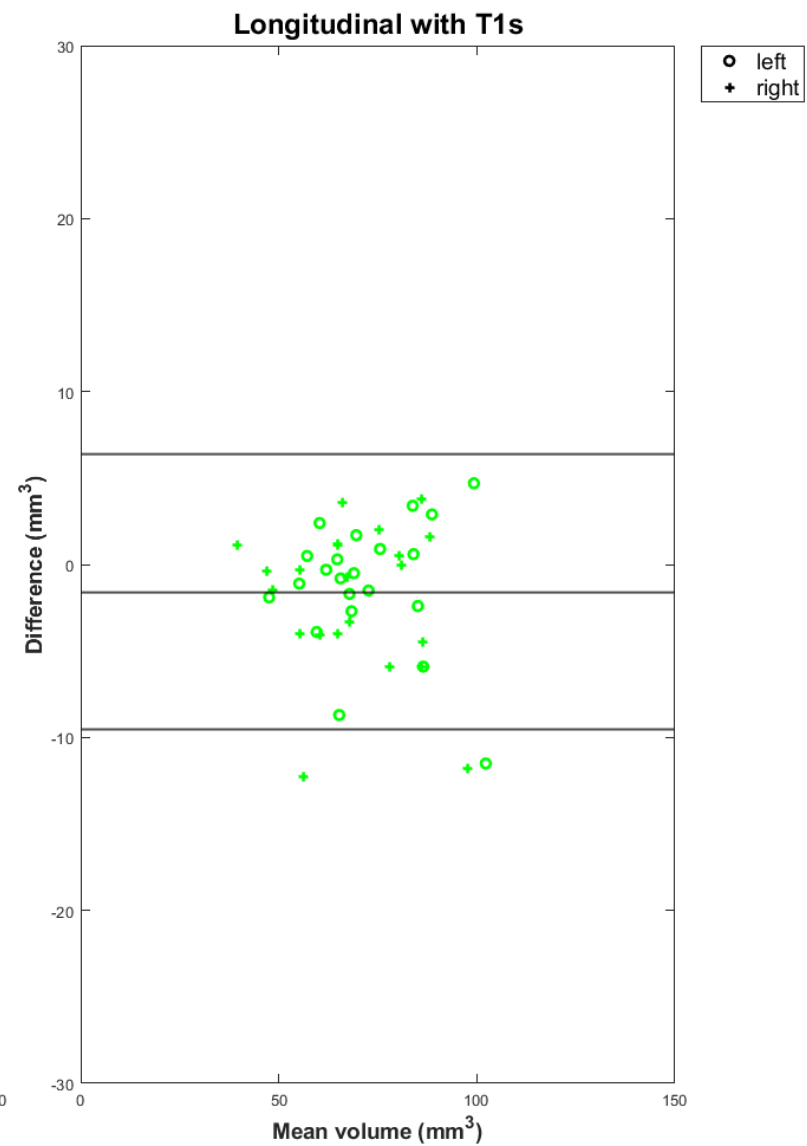

# HATA

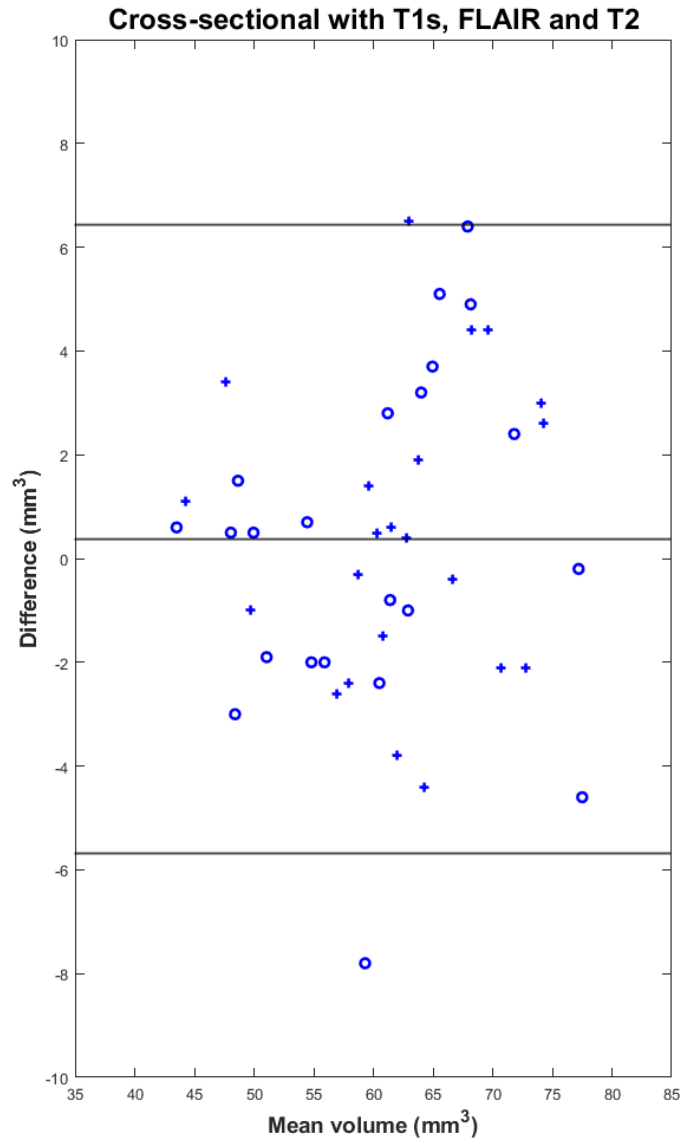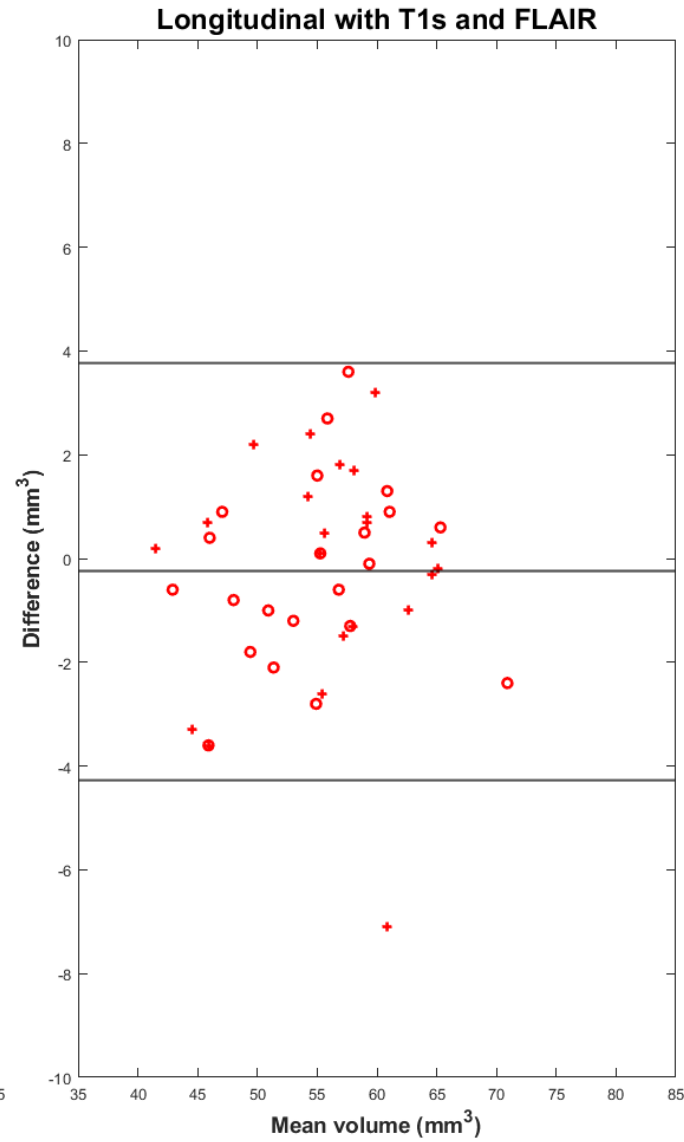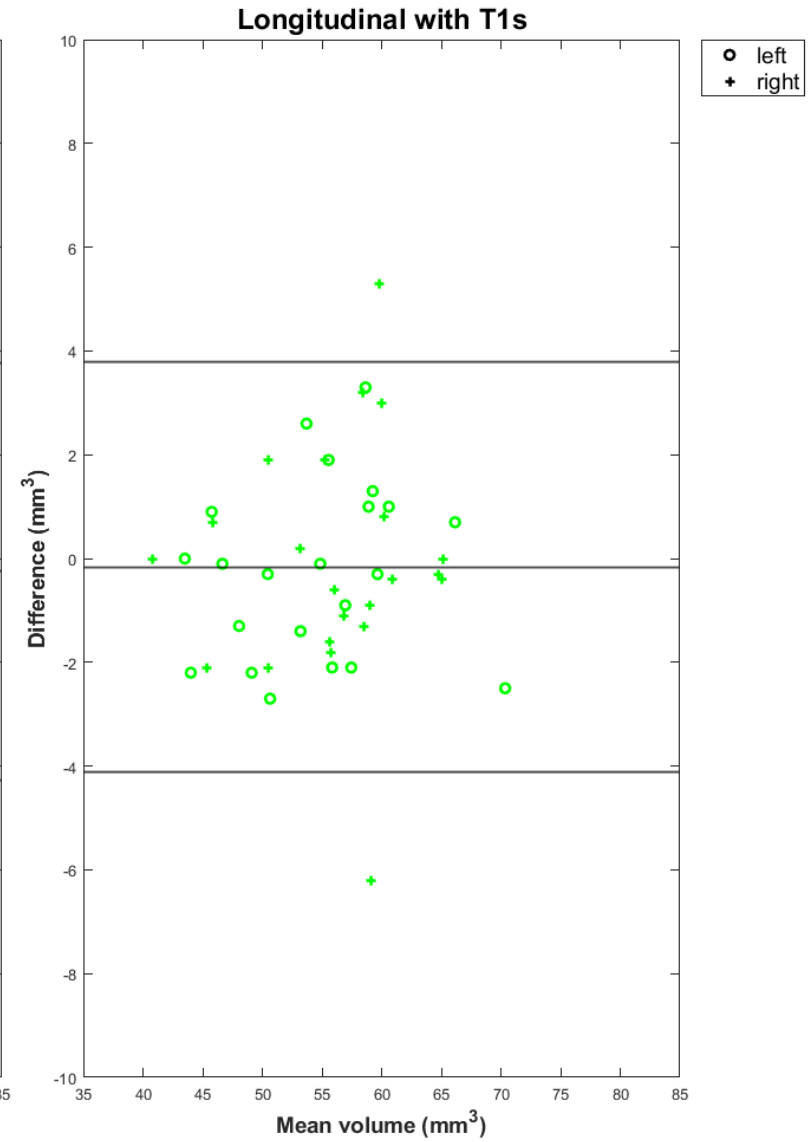

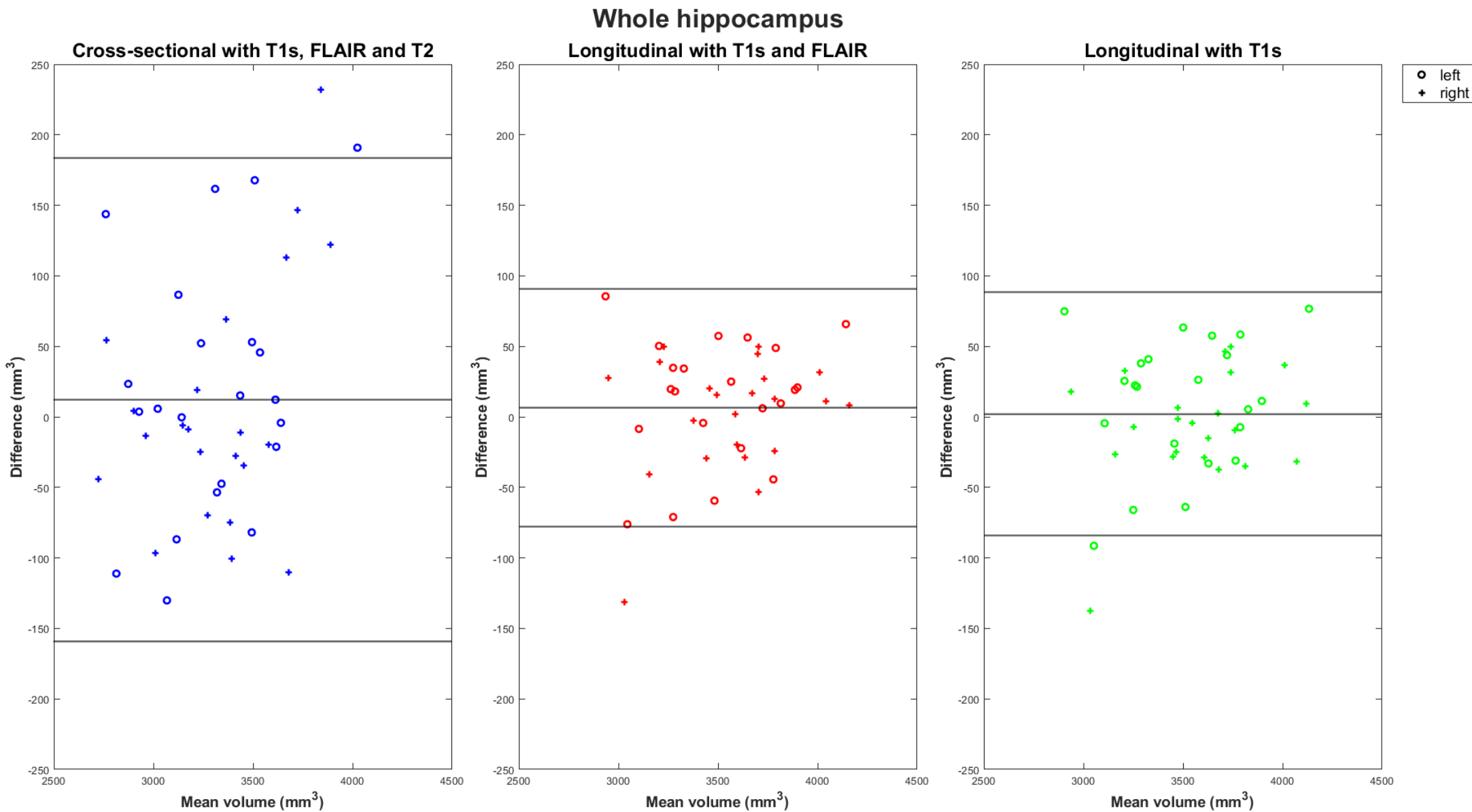

## Whole hippocampus + fissure

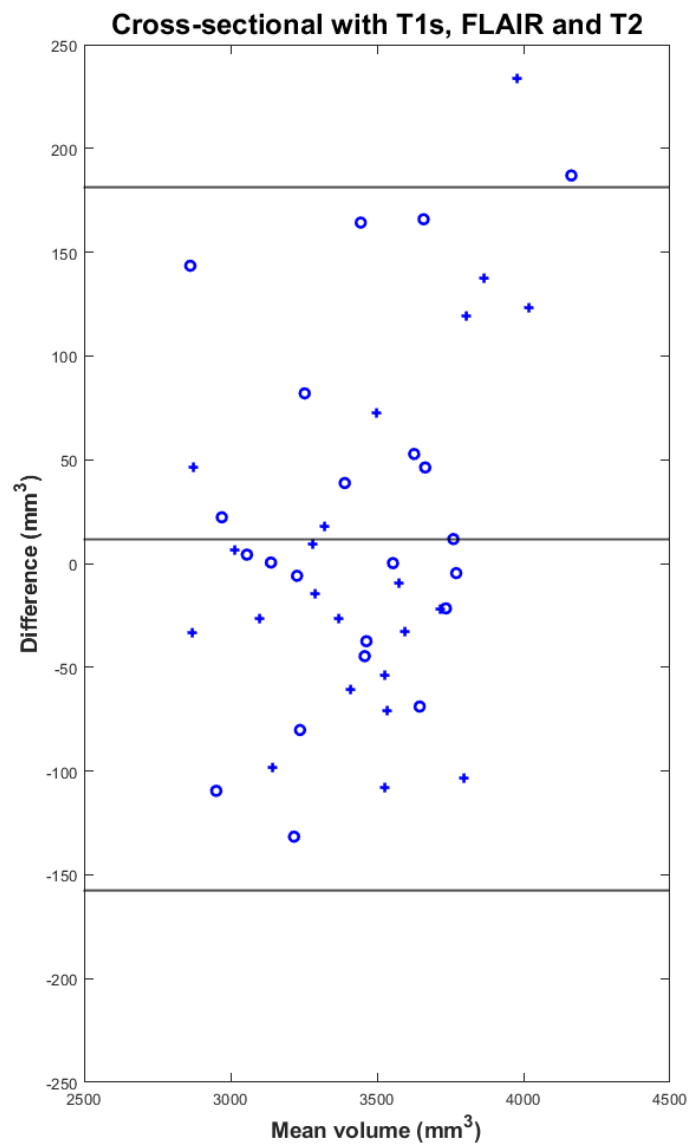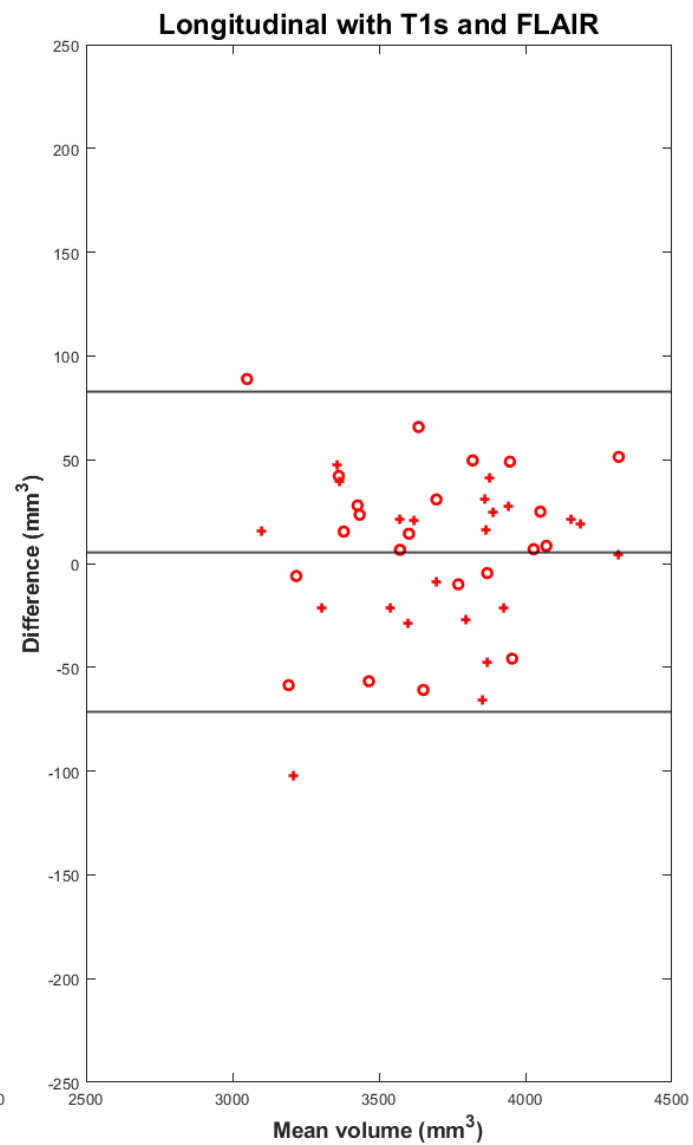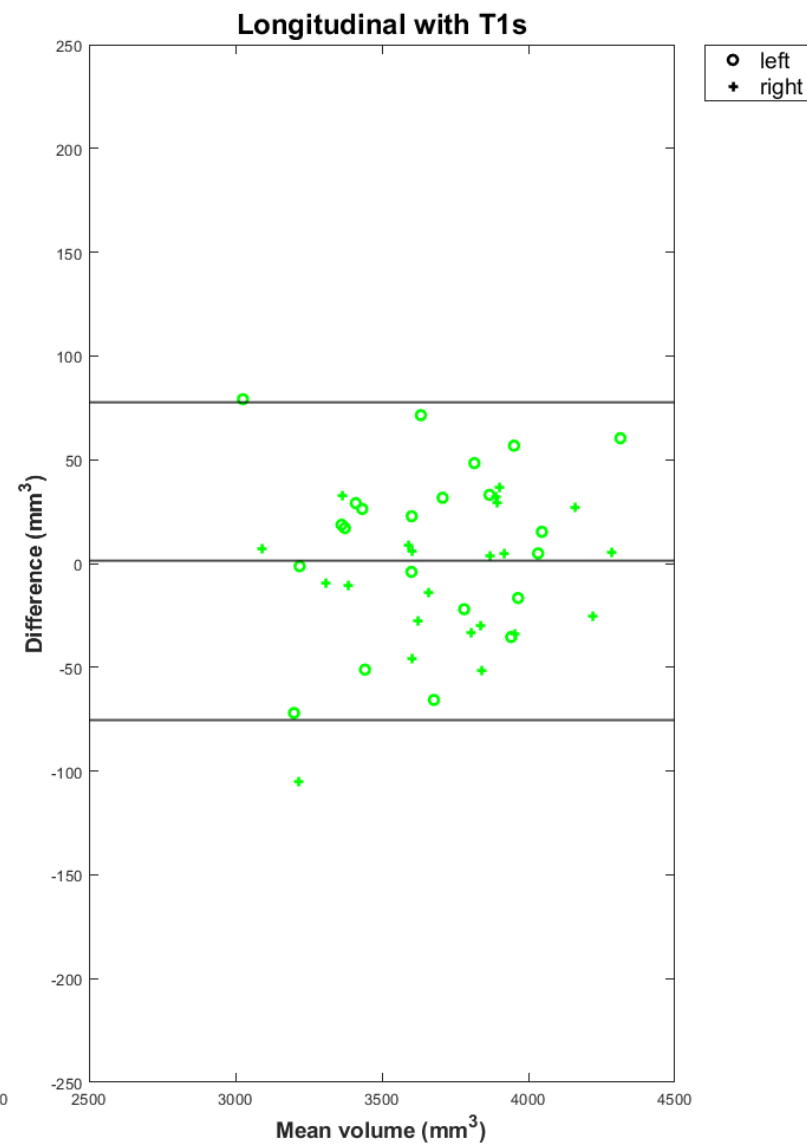

Supplement: Supplementary file 1 — Suppl. Fig. 1 Bland-Altman plots showing test-retest volume differences ofhippocampal structures as a function of mean structure volume for each segmentationpipeline (long_T1s, long_T1s_FLAIR and long_T1s_FLAIR_crossT2). For eachstructure and pipeline, the plot shows the test-retest structure mean volume and testretestdifference for each subject (left hemisphere: circles, right hemisphere: plus sign),with the mean volume difference and the 95% confidence intervals (solid lines) (PDF 1184 KB) [file 429_2020_2172_MOESM1_ESM.pdf]
